# Human and Mouse Alzheimer's Seeds Differentially Affect Amyloid Deposition and Microglia‐Dependent Plaque Response in Aged Mice

**DOI:** 10.1111/acel.70094

**Published:** 2025-05-13

**Authors:** Juana Andreo‐Lopez, Cristina Nuñez‐Diaz, Kelly Do Huynh, Marie Minh Thu Nguyen, Celia Da Cunha, Francisco J. Cantero‐Molina, Cynthia Campos‐Moreno, Stefania Zimbone, Francesco Bellia, Maria Laura Giuffrida, Laura Trujillo‐Estrada, Juan Antonio Garcia‐Leon, Miriam Bettinetti‐Luque, Nazaret Gamez, Catalina Valdes, Rodrigo Morales, Stefania Forner, Alessandra C. Martini, Antonia Gutierrez, Frank M. LaFerla, David Baglietto‐Vargas

**Affiliations:** ^1^ Departamento de Biologia Celular, Genetica y Fisiologia Instituto de Investigacion Biomedica de Malaga y Plataforma en Nanomedicina‐IBIMA Plataforma BIONAND, Facultad de Ciencias, Universidad de Malaga Malaga Spain; ^2^ CIBER de Enfermedades Neurodegenerativas (CIBERNED) Instituto de Salud Carlos III Madrid Spain; ^3^ Institute for Memory Impairments and Neurological Disorders University of California Irvine California USA; ^4^ Institute of Crystallography National Research Council (CNR‐IC) Catania Italy; ^5^ Department of Biomedical and Biotechnological Sciences University of Catania Catania Italy; ^6^ Department of Neurology The University of Texas Health Science Center at Houston Houston Texas USA; ^7^ Centro Integrativo de Biologia y Quimica Aplicada Universidad Bernardo O'Higgins Santiago Chile; ^8^ Department of Neurobiology and Behavior University of California Irvine California USA

**Keywords:** Alzheimer's disease, amyloid‐beta, inflammation, propagation, seeds, tau, transgenic mice

## Abstract

Alzheimer's disease (AD) is a complex neurodegenerative proteinopathy in which Aβ and tau misfold and aggregate into entities that structurally unsettle native proteins, mimicking a prion‐like or “seeding” process. These Aβ and tau “seeds” can arrange in different conformations or strains that might display distinct pathogenic properties. Furthermore, recent evidence suggests that microglia play a key role in the amyloidogenic event and can modulate the propagation and aggregation processes. Here, we employed histological and molecular approaches to determine whether seeds from human AD brains compared to those from transgenic mice (3xTg‐AD) are more prone to induce Aβ and tau aggregates in vivo, as well as potential differences in the microglial response to the plaque pathology. Brain homogenates were injected into the hippocampus of 3xTg‐AD mice and hAβ‐KI mice and examined at 18–20 months of age. The seeds from the human AD brain induced more aggressive amyloid pathology compared to seeds from aged 3xTg‐AD mice. However, the AD seeds from aged transgenic mice triggered more tau pathology. Interestingly, such mice seeds impaired microglial clustering around plaques, leading to more severe neuritic pathology. Furthermore, the human AD seeds injected into the hippocampus of hAβ‐KI mice were not able to induce plaque formation. These results suggest that multiple variables such as the AD seed, recipient model, and time are critical factors that can modulate the amyloid pathology onset and progression. Thus, more profound understanding of these factors will provide key insight into how amyloid and tau pathology progresses in AD.

## Graphical abstract

This study shows that human and mouse brains contain a different pool of strains that differentially affect the formation of Aβ and tau aggregates. Moreover, the murine seeds trigger an unresponsiveness microglial state, which causes the loss of plaque microglial coverage and affects the formation of amyloid plaques, leading to more severe neuritic pathology. The findings provide insights into the differences between mouse and human Aβ and tau strains and their impact on disease progression.

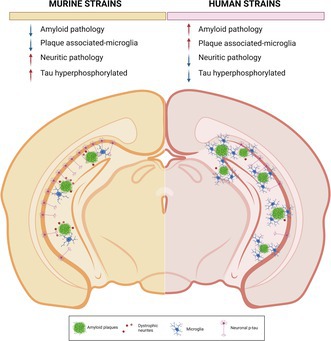

**Abbreviations**
AD: Alzheimer's disease
APOE4: Apolipoprotein E4
APP: amyloid precursor protein
Aβ: amyloid‐β
Aβ_N3pE_
: Pyroglutamate Aβ
Aβp_Ser8_
: Phosphorylated Aβ
BSA: bovine serum albumin
CD45: cluster of differentiation 45
CD68: cluster of differentiation 68
DAB: 3‐3‐DiaminoNenzidine tetrahydrochloride
ELISA: enzyme‐linked immunosorbent assay
EOAD: early‐onset Alzheimer's disease
GFAP: glial fibrillary acidic protein
HFIP: 1,1,1,3,3,3‐hexafluoro‐2‐propanol
HRP: horseradish peroxidase
Iba1: ionized calcium binding adaptor molecule 1
LC–MS: liquid chromatography–mass spectrometry analysis
LOAD: late‐onset Alzheimer's disease
MS: mass spectrometry
MTT: 3‐(4,5‐dimethylthiazol‐2‐yl)‐2,5‐diphenyltetrazolium bromide
NFTs: neurofibrillary tangles
NIH: National Institutes of Health
Old‐Tg mice: old 3xTg‐AD mice
P2RY12: purinergic receptor P2Y12
PAS: periodic acid schiff
PB: phosphate buffer
PBS: phosphate buffered saline
PMCA: protein misfolding cyclic amplification
p‐tau: phosphorylated‐tau
SEM: standard error of the mean
ThioS: thioflavin‐S
ThT: thioflavin‐T
TIMs: terminally inflammatory microglia
TREM2: triggering receptor expressed on myeloid cells 2
UCI MIND: Institute of Memory Impairment and Neurological Disorders
V: volume
W: weight
WB: western‐blot
XIC: extracted ion chromatogram

## 1 Introduction

Many neurodegenerative disorders, including Alzheimer's disease (AD), frontotemporal dementia, Parkinson's disease, Lewy bodies dementia, and amyotrophic lateral sclerosis, have in common the accumulation and aggregation of pathogenic proteins such as amyloid‐β (Aβ), phospho‐tau (p‐tau), α‐synuclein, or TAR DNA‐binding protein 43 (Brettschneider et al. 2015; Jucker and Walker 2013, 2018). Once misfolded, these proteins can act as pathogenic seeds, mimicking prion‐like mechanisms and inducing an aberrant or alternative conformation to native proteins. Such alternatively folded proteins are usually organized in β‐sheet conformations and tend to form aggregates that spread through specific brain structures, distressing their normal function and causing multiple detrimental symptoms in the affected individuals (Brettschneider et al. 2015; Jucker and Walker 2013, 2018).

Alzheimer's disease is the most prevalent brain proteinopathy, with currently nearly 50 million people affected worldwide, and the number of cases is projected to exceed 100 million by 2050 (GBD Dementia Forecasting Collaborators 2022). Histologically, AD has been characterized by two pathological hallmarks: extracellular amyloid plaques and intracellular neurofibrillary tangles (NFTs). Both result from the accumulation and aggregation of misfolded proteins, in this case the Aβ peptide and hyperphosphorylated tau, respectively, which end up originating insoluble deposits (Iadanza et al. 2018; Li and Liu 2022; Soto and Pritzkow 2018; Subedi et al. 2022). In addition, this neurodegenerative disorder courses with synaptic and neuronal loss, glial activation/dysfunction, and metabolic disturbances that are also associated with the accumulation of toxic protein aggregates (Long and Holtzman 2019; Soto and Pritzkow 2018).

Since the initial studies demonstrating the self‐propagation properties of Aβ (Baker et al. 1993; Goudsmit et al. 1980; Jarrett and Lansbury Jr. 1992; Kane et al. 2000), multiple reports have shown that both proteins, Aβ and tau, can act as transmissible agents, mimicking prion‐like mechanisms of propagation (Brettschneider et al. 2015; Jucker and Walker 2013, 2018). From these pioneering studies, we have learned that (i) brain extracts from human or rodent donors contain Aβ seeds with higher seeding efficiencies compared with synthetic Aβ aggregates, (ii) these brain extracts and also synthetic/recombinant aggregates can propagate Aβ or tau pathology following intracerebral or peripheral administrations, (iii) heterologous seeding (cross‐seeding) can occur between different amyloid proteins, (iv) Aβ seeds can be transmitted through the vascular system, (v) different genetic risk variants such as the apolipoprotein E4 (APOE4) modulate Aβ and tau propagation, and (vi) tau is also propagated via the transcellular pathway (Bu et al. 2018; Chen et al. 2024; Eisele et al. 2010; Friesen and Meyer‐Luehmann 2019; He et al. 2018; Jaunmuktane et al. 2015; Kaufman et al. 2018; Liu et al. 2017; Meyer‐Luehmann et al. 2006; Morales et al. 2021; Moreno‐Gonzalez et al. 2017). In general, these studies highlight the importance of the multiple factors that are involved in these mechanisms, and the processes that are critical for the development and propagation of Aβ and tau aggregates.

On the other hand, microglial cells have been reported to play a central role in plaque formation, spreading, and propagation (d'Errico et al. 2022; Pascoal et al. 2021). Multiple studies demonstrate that these glial cells form a barrier surrounding the amyloid plaques and attenuate their dissemination, thereby reducing neuronal damage (Chen and Colonna 2022; Sosna et al. 2018; Spangenberg et al. 2019). However, new intriguing finding also indicates that microglia is key for spreading Aβ aggregates across different brain areas by releasing phagocytosed Aβ fragments from pathogenic to nonpathogenic regions, suggesting that microglia displays an important role guiding the dissemination of the Aβ seeds (Chen and Colonna 2022; d'Errico et al. 2022; Spangenberg et al. 2019). A similar effect may also occur with tau pathology (Asai et al. 2015; Chen et al. 2024; Gratuze et al. 2021; Hopp et al. 2018; Leyns et al. 2019; Maphis et al. 2015; Wang et al. 2022) and interestingly, it has been recently reported that microglial activation anatomically correlates with tau pathology Braak staging in AD brains (Pascoal et al. 2021).

Overall, most of these studies have been performed in amyloidogenic or tauopathy models that overproduce human amyloid precursor protein (APP) with mutations associated with familial AD or overexpressing mutated forms of tau. However, it is imperative to study these pathogenic seeding and propagation processes in more physiological models that mimic sporadic forms of AD. For this reason, here we used a sporadic AD model, the human Aβ knock‐in mice, or hAβ‐KI, developed and characterized by the MODEL‐AD consortium at University of California (Baglietto‐Vargas et al. 2021). In this current study, by stereotaxically injecting different brain homogenates containing AD‐linked seeds, we investigated how Aβ misfoldings propagate in two relevant animal models representative of the familial (3xTg‐AD) and sporadic (hAβ‐KI) forms of brain amyloidosis. In addition, we also determined the impact of such inocula on tau propagation. In general, the present study shows that the human AD homogenate used in this study contains different conformational amyloid strains that stimulate the formation of amyloid plaques more rapidly than those found in aged 3xTg‐AD brain samples. On the contrary, tau pathology is more efficient after the injection of aged 3xTg‐AD brain extract compared to the human AD sample. In addition, aged 3xTg‐AD mice seeds trigger a significant reduction of microglial cells clustering around amyloid plaques and an increment of neuritic pathology. Finally, different amyloid propagation rates occur in 3xTg‐AD mice compared to the hAβ‐KI, probably taking a quite longer time to possibly generate Aβ plaques in this late onset‐AD animal model.

## 2 Material and Methods

### 2.1 Transgenic Mice

Female 3xTg‐AD mice, and male and female hAβ‐KI and APP/PS1 mice, all previously described (Baglietto‐Vargas et al. 2021; Oddo et al. 2003), were used. Briefly, the 3xTg‐AD line was generated by the microinjection of two human transgenes (APP_swe_ and MAPT_P301L_, both under the control of the mouse Thy1.2 promoter) into single‐cell embryos from mice knock‐in for the PS1_M146V_ gene. The hAβ‐KI model was generated by homologous recombination in mouse embryonic stem cells, in which the G676R (G5R), F681Y (F10Y) and R684H (R13H) mutations were introduced into the endogenous *App* gene. APP/PS1 mice express the human APP including the Swedish mutation and the delta E9 mutation in the presenilin 1 (Jankowsky et al. 2001). All animal experiments were carried out in accordance with the National Institutes of Health (NIH) and with the Spanish and the European Union regulations (RD53/2013 and 2010/63/EU) approved by the local Animal Research Committees from the University of California (USA), University of Malaga (Spain) and The University of Texas Health Science Center at Houston (USA). Experiments and procedures with animals were designed to minimize animal suffering and reduce the number of animals used.

### 2.2 Human Tissue

Human *postmortem* AD prefrontal cortex was provided by the Institute of Memory Impairment and Neurological Disorders (UCI MIND) from the University of California (Irvine, CA, USA). The tissue was obtained at autopsy (*N* = 1, 77 years old female case; Braak VI stage for tau pathology and C stage for plaque pathology) with a *postmortem* delay time of 4 h (Figure S1A). Additional prefrontal cortex from an AD patient (79 years old female) and a cognitively normal individual (79 years old female) displaying AD neuropathology were provided by the National Disease Resource Interchange (NDRI). The utilization of the human brain samples was approved by the corresponding ethic committee at the University of California, Irvine, and The University of Texas Health Science Center at Houston (USA).

### 2.3 Brain Homogenates and Intracerebral Stereotaxic Injections

**FIGURE 1:**
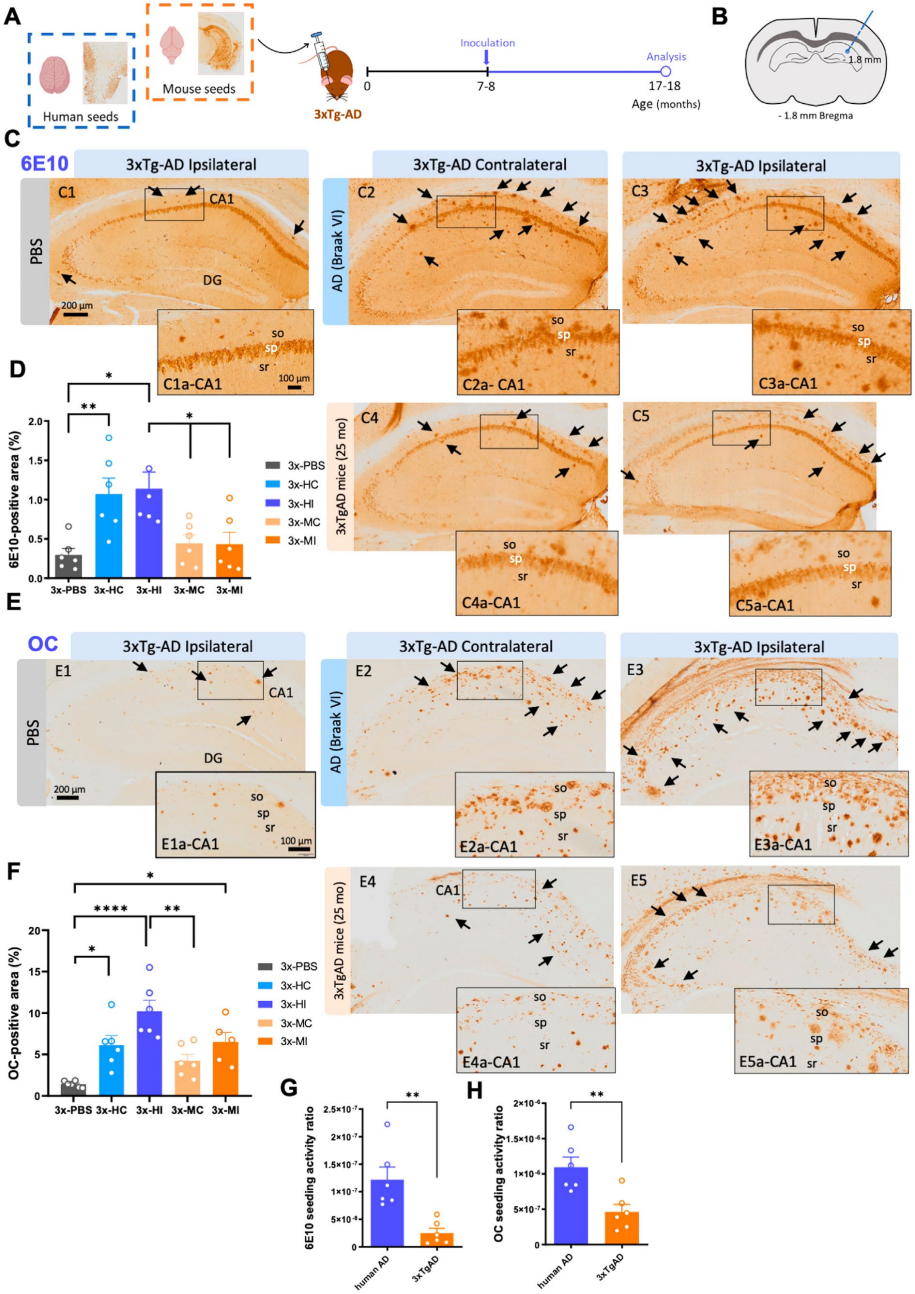
Human and transgenic mice AD seeds differentially accelerate amyloid pathology in 3xTg‐AD. (A) Brain homogenates from an AD patient and an old 3xTg‐AD mice (25 months) were inoculated into the hippocampus of 7–8‐month‐old female 3xTg‐AD mice, followed by a 10‐month incubation period. (B) Stereotaxic injections were performed into the right hemisphere of the hippocampus (AP −1.8 mm, ML −1.8 mm, DV −1.8 mm), represented by a blue needle outline. Exactly 2.5 μL of inoculum were deposited in each mouse brain, whether it was PBS, old‐Tg mice or AD patient's homogenate. (C) Hippocampal microscopic images immunolabeled for 6E10 in 3xTg‐AD mice treated with PBS (C1, C1a) and with brain homogenates from AD patient in the contralateral (C2, C2a) and ipsilateral (C3, C3a) sides, and with brain samples from old 3xTg‐AD mice in the contralateral (C4, C4a) and ipsilateral (C5, C5a) sides. (D) Quantification of Aβ load with 6E10 in the ipsilateral and contralateral side of the 3xTg‐AD treated with human brain homogenates compared with old‐Tg mice seeds and PBS. (E) Hippocampal microscopic images stained with OC in 3xTg‐AD mice treated with PBS (E1, E1a) brain homogenates from AD patient in the contralateral (E2, E2a) and ipsilateral (E3, E3a) sides, and with brain samples from old 3xTg‐AD mice in the contralateral (E4, E4a) and ipsilateral (E5, E5a) sides. (F) Quantification of the OC burden of brain slices in the ipsilateral and contralateral side of the 3xTg‐AD treated with PBS, human brain homogenates and old‐Tg mice seeds. (G) Activity ratio graph of the ipsilateral side of 3xTg‐AD mice treated with human AD brain homogenate compared to old‐Tg mice samples. (H) Activity ratio graph of the ipsilateral side of 3xTg‐AD mice treated with human AD brain homogenate compared to old‐AD mice samples. C, contralateral; DG, dentate gyrus; H, human; I, ipsilateral; M, mouse; so, *Stratum oriens*; sp., *Stratum piramidale*; sr, *Stratum radiatum*. The values represent means ± SEM. *N* = 5–6 per group. Scale bar: 200 μm (C1–C5 and E1–E5) and 100 μm (C1a–C5a and E1a–E5a).

Murine brain homogenates were obtained from 25‐month‐old 3xTg‐AD mice (old‐Tg mice) that were perfused transcardially with 0.1 M phosphate buffered saline (PBS, pH 7.4) for 3 min. Human and mice brain samples were homogenized in sterile PBS (pH 7.4) at 10% (w/v) and sonicated with 3 pulses of 5 s (Branson 450 Sonifier). The crude brain homogenates were centrifuged for 5 min (at 3000 × *g*, 4°C) and the supernatants (brain extracts) stored at −80°C until use. Six‐ to seven‐month‐old 3xTg‐AD and hAβ‐KI mice (*N* = 5–6 per group) were anesthetized with isoflurane and unilateral stereotaxic injections of old‐Tg mice cortical brain extract or human AD extract (2.5 μL at an injection speed of 1.25 μL/min) were performed into the right hemisphere of the hippocampus (coordinates from Bregma: AP −1.8 mm, ML −1.8 mm, DV −1.8 mm) and overlying neocortex (coordinates AP −1.8 mm, ML −1.8 mm, DV −1.0 mm; in this region 1 μL was inoculated) (Figure 1A,B). Another set of mice (3xTg‐AD) were injected with PBS as control animals. After each injection, the Hamilton syringe needle was kept in place for an additional 2.5 min before being slowly withdrawn. The surgical area was cleaned with sterile saline, the incision was sutured, and the mice were monitored until recovery from anesthesia. Animals were sacrificed at 10 months postsurgery (Figure 1A).

For another set of mice (APP/PS1 and hAβ‐KI mice), human samples were prepared by homogenization in an automatic homogenizer (Precellys) following the manufacturer's recommendations. These were prepared at 10% w/v in PBS supplemented with a protease inhibitor cocktail (Roche Diagnostics GmbH, Germany), vortexed, aliquoted, and stored at −80°C until use (no sonication or centrifugation). hAβ‐KI mice (*N* = 15; 53% males for the NDAD sample, and *N* = 17; 47% female for the AD sample) were injected with these materials at 3 months old and sacrificed at 20 months old (17 months incubation period). APP/PS1 mice (*N* = 6, 67% male for NDAD, and *N* = 6, 50% female for the AD sample) were injected with the same samples at 2 months old and sacrificed at 6 months old. Injections were performed as described above, with the sole difference that they were conducted bilaterally, using 10 μL of brain extract in each injection.

### 2.4 Electrochemiluminescence‐Linked Immunoassay

Amyloid‐beta levels were measured by using the V‐Plex Aβ peptide panel 1 kit according to the manufacturer's instructions (Meso Scale Discovery, Rockville, MD, USA; Catalog #K15200E‐1). Briefly, 3xTg‐AD mice and human samples were loaded onto the electrochemiluminescence‐linked immunoassay plate. Standards (including Aβ1‐38, Aβ1‐40 and Aβ1‐42) and samples were added to the 96‐well plate and incubated overnight, washed, and read in a Sector Imager plate reader MSD MESO QuickPlex SQ120 (Meso Scale Discovery) immediately after the addition of the MSD read buffer. Aβ concentration was calculated with reference to the standard curves and expressed as micrograms per milligram of protein.

### 2.5 Enzyme‐Linked Immunosorbent Assay

Human total tau levels were measured by using the enzyme‐linked immunosorbent assay (ELISA) kit according to the manufacturer's instructions (ThermoScientific, Rockford, IL, USA; Catalog #KHB0041). Briefly, standards, 3xTg‐AD mice, and human AD samples were prepared and loaded onto the 96‐well plate and incubated for 2 h. Then, they were washed and proceeded to the binding of the biotin, followed by the streptavidin conjugated to the horseradish peroxidase (HRP) and then the stabilized chromogen. Washes were included in every indicated step. Finally, the stop solution was added, and the absorbance was read at 450 nm in a spectrophotometer. Human total tau concentration in every sample was calculated with reference to the standard curve and expressed as picograms per milliliter.

### 2.6 Dot‐Blot and Western‐Blot

For the western‐blot (WB) assays, equal amounts of protein (10 μg) were fractionated on NuPAGE 10% Bis‐Tris Gel (Invitrogen, Waltham, MA, USA; Catalog #NP0302BOX) and transferred to a nitrocellulose membrane (Bio‐Rad, Hercules, CA, USA; Catalog #1704159). For the dot blot assay, protein‐normalized samples (1 μg total protein) were directly loaded onto 0.2 μm nitrocellulose membranes (ThermoScientific, USA; Catalog #88024). Next, membranes were blocked for 1 h at room temperature in a solution with 5% w/v bovine serum albumin (BSA; Sigma‐Aldrich, Madrid, Spain; Catalog #A9418) and incubated overnight at 4°C with one of the following primary antibodies: 6E10 (mouse monoclonal against Aβ1‐16, 1:1000; BioLegend, Amsterdam, The Netherlands; Catalog #SIG‐39320), A11 (rabbit, 1:1000; Millipore, Madrid, Spain; Catalog #AB9234), AT8 (mouse monoclonal, 1:1000; ThermoScientific; Catalog #MN1020), OC (rabbit, 1:1000; Millipore; Catalog #AB2286), HT7 (mouse monoclonal, 1:1000; Invitrogen; Catalog #MN1000), MC1 (mouse monoclonal, 1:500; provided by Dr. Peter Davies, Albert Einstein College of Medicine, Manhasset, NY, USA) or PHF1 (mouse monoclonal, 1:500; provided by Peter Davies, Albert Einstein College of Medicine, Manhasset, NY, USA). The membranes were then washed and incubated for 1 h at room temperature with a specific secondary antibody: goat anti‐mouse HRP conjugate (1:3000; Bio‐Rad; Catalog #1706516) or goat anti‐rabbit HRP conjugate (1:3000; Bio‐Rad; Catalog #1706515). Membranes were developed using SuperSignal West Pico PLUS Chemiluminescent Substrate (ThermoScientific; Catalog #34580) and quantified using the image analysis software ImageJ (v1.53c) (National Institutes of Health freeware). For the WB normalization, β‐tubulin was used as a loading control (rabbit anti‐β‐tubulin antibody; 1:2500; Cell Signaling, Danvers MA, USA; Catalog #2146S; and the specific goat anti‐rabbit HRP conjugate secondary antibody (1:3000; Bio‐Rad; Catalog #1706515)).

### 2.7 Liquid Chromatography‐Mass Spectrometry Analysis

All the samples were lyophilized and resuspended in 1,1,1,3,3,3‐hexafluoro‐2‐propanol (HFIP) to dissolve the aggregated forms of the Aβ peptide. The mixtures were then sonicated for 15 min and incubated at 37°C for 45 min. After centrifugation (5 min at 12,000 x *g*), HFIP was removed under a stream of nitrogen. Samples were resuspended in phosphate buffer (50 mM, pH 7.4) and treated with trypsin (enzyme/protein 1:20 w:w) at 37°C for 20 h. The peptide content was analyzed by liquid chromatography‐mass spectrometry (LC–MS), using the Ultimate 3000 HPLC RSLCnano system coupled to a hybrid quadrupole‐Orbitrap mass spectrometer (Q‐Exactive, Thermo Scientific, Milan, Italy) through an EASY‐Spray source (Thermo Scientific, Milan, Italy). The technical parameters were set up as previously reported (Falcone et al. 2020). The extracted ion chromatogram (XIC) due to each charged species of the hydrolytic peptide fragment was used for peak detection. Both the high‐resolution mass spectrometry (MS) spectra of these species and the related MS/MS spectra were useful in identifying the peptide fragments. The 6–16 and 17–18 tryptic peptides were used to quantify human‐Aβ in the samples.

### 2.8 Aβ and Tau Immunodepletion Assay

For the immunodepletion of Aβ and tau peptides from the brain extracts, the Dynabeads Protein G Immunoprecipitation Kit (Invitrogen; Catalog #10007D) was used according to the manufacturer's guide. In brief, 4.5 mg of Dynabeads magnetic beads were washed and incubated with 10 μg of the antibody of interest, being 6E10 (mouse monoclonal against Aβ1‐16; BioLegend; Catalog #SIG‐39320) for Aβ immunodepletion, and HT7 (mouse monoclonal antibody against human tau between residue 159 and 163; ThermoScientific; Catalog #MN1000) in combination with AT8 (mouse monoclonal against phospho‐tau in the residue Ser202 and Thr205; ThermoScientific; Catalog #MN1020) for tau immunodepletion (5 μg of each), as well as the three antibodies combined together to perform the Aβ and tau simultaneous immunodepletion. Once the magnetic beads were attached to the antibodies, these particles of interest were captured using a magnet (DynaMag‐2; Invitrogen; Catalog #12321D) and further incubated overnight at 4°C to avoid unspecific binding with the brain homogenates to capture the Aβ and tau proteins. Finally, beads with the bound proteins of interest were captured again and the remaining immunodepleted homogenates were used to treat the BV‐2 cells. Accordingly, the captured proteins were eluted in the elution buffer and subsequently run in a WB with the immunodepleted extracts to confirm successful immunodepletion (Figure S4A).

### 2.9 MTT Assay

Viability of BV‐2 cells was tested by using the 3‐(4,5‐dimethylthiazol‐2‐yl)‐2,5‐diphenyltetrazolium bromide (MTT) assay according to the manufacturer's instructions (Sigma‐Aldrich; Catalog #M2128). BV‐2 cells were seeded into a 96‐well plate at a concentration of 1.7 × 10^3^ cells/well. After 3 h, AD seeds‐loaded brain extracts from human AD patients and old‐3xTg‐AD mice, or immunodepleted homogenates, were added to the wells. After incubation of the BV‐2 cells with such seeds for 72 h at 37°C, MTT (5 mg/mL) was added to each well and further incubated for 4 h at 37°C. Next, medium was removed and acid isopropanol (HCl 0.04 N) was added to dissolve the formed formazan crystals. Finally, absorbance was read at 550 nm.

### 2.10 ThT Assay

In order to study the effect of seeds from brain homogenates on the amyloid‐type aggregation of Aβ, we carried out an assay using a protocol previously reported (Greco et al. 2020). Aβ1‐40 was properly treated with HFIP (Greco et al. 2020), in order to enrich the sample of the monomer species. HFIP‐treated Aβ1‐40 was then dissolved in 10 mM NaOH and diluted (2 μM) in PBS containing Thioflavin‐T (ThT) (6 μM) (LeVine 3rd 1993), with or without brain homogenates (0.1%) coming from old 3xTg‐AD mice and human AD patients (*N* = 4). Samples were incubated at 37°C for 24 h, and the fluorescence measurements (excitation and emission wavelengths were 450 nm and 480 nm, respectively) were carried out in triplicate using the multi‐plate reader Victor Nivo (Milan, Italy). The collected fluorimetric data were fitted to Equation (1).

(1) $$F\left(t\right)=\frac{F_{\max}-F_{0}}{1+e^{-\frac{t-t_{½}}{k}}}$$

*F*_*max*_ *—F*_*0*_ is the maximum fluorescence increment during the amyloid‐type aggregation value. This value for all the samples has been reported as a percentage of that measured in the seed‐free Aβ sample. The lag phase (*t*_*lag*_), that is the time period before the formation of amyloid species, can be calculated by using Equation (2).

(2) tlag=t½−2k

The parameters of each set of measurements were expressed as means ± standard error of the mean (SEM).

### 2.11 Immunolabeling and Histological Stains

After deep sedation with CO_2_, mice were perfused transcardially with 4% paraformaldehyde in 0.1 M phosphate buffer (PB, pH 7.4). Brains were postfixed overnight, cryoprotected in 30% sucrose, sectioned at 40 μm thickness in the coronal plane on a freezing microtome, and serially collected as free‐floating sections.

For light microscopy immunostaining, sections were first treated with 3% hydrogen peroxide and methanol in PBS pH 7.4 for 20 min to block endogenous peroxidase activity. Next, slices were incubated with avidin‐biotin blocking kit (Vector Labs, Newark, CA, USA; Catalog #SP‐2001) for 30 min and 5% BSA (Sigma‐Aldrich) in PBS for 1 h at room temperature to avoid unspecific binding. Immunolabeling was performed using one of the following antibodies: 4G8 (mouse monoclonal, 1:2000; BioLegend; Catalog #800704), 6E10 (mouse monoclonal, 1:5000; BioLegend; Catalog #SIG‐39320), Aβ40 (rabbit polyclonal, 1:2000, Millipore; Catalog #AB5074P), Aβ42 (rabbit polyclonal, 1:2000, Millipore; Catalog #AB5078P), AT8 (mouse monoclonal, 1:1000; ThermoScientific; Catalog #MN1020), MC1 (mouse monoclonal, 1:500; provided by Peter Davies, Albert Einstein College of Medicine, Manhasset, NY, USA), PHF1 (mouse monoclonal, 1:500; provided by Peter Davies, Albert Einstein College of Medicine, Manhasset, NY, USA) and OC (rabbit polyclonal, 1:5000, Millipore; Catalog #AB2286). Sections were then incubated with anti‐mouse (1:500, Vector Labs) or anti‐rabbit (1:500, Vector Labs) biotinylated secondary antibodies in PBS for 75 min at room temperature. Next, samples were treated with streptavidin‐conjugated horseradish peroxidase for 90 min (1:2000, Sigma‐Aldrich; Catalog #GERPN1231‐2ML) before visualization with 0.05% 3‐3‐diaminobenzidine tetrahydrochloride (DAB; Sigma‐Aldrich; Catalog #D7304‐1SET) and 0.01% hydrogen peroxide in PBS. Microscopy images were obtained with an Olympus VS120 slide scanner.

For immunofluorescence labeling, sections were incubated with 5% BSA PBS (Sigma‐Aldrich) for 1 h at room temperature, followed by a 24‐h incubation with one or two of the following primary antibodies: 6E10 (mouse monoclonal, 1:5000; BioLegend; Catalog #SIG‐39320), APP (rabbit polyclonal, 1:10000; Sigma‐Aldrich; Catalog #A8717), AT8 (mouse monoclonal, 1:1000; ThermoScientific; Catalog #MN1020), CD45 (rat polyclonal, 1:500; Bio‐Rad; Catalog #MCA1388), CD68 (rabbit polyclonal, 1:1000; Abcam Cambridge, UK; Catalog #AB125212), GFAP (rabbit polyclonal, 1:2000; Dako, Santa Clara, CA, USA; Catalog #Z0334), Iba1 (rabbit polyclonal, 1:1000; Wako, Osaka, Japan; Catalog #019–19,741), Iba1 (goat polyclonal, 1:1000; Abcam; Catalog #ab5076), MC1 (mouse monoclonal, 1:500; provided by Peter Davies, Albert Einstein College of Medicine, Manhasset, NY, USA), P2RY12 (rabbit polyclonal, 1:200; Sigma‐Aldrich; Catalog #HPA014518), PHF1 (mouse monoclonal, 1:500; provided by Peter Davies, Albert Einstein College of Medicine, Manhasset, NY, USA) or TREM2 (sheep polyclonal, 1:1000; R&D Systems, Abingdon, UK; Catalog #AF1729). Next, sections were incubated for 1 h in the corresponding secondary antibody conjugated to Alexa fluorochrome (1:1000; Invitrogen): donkey anti‐goat 488 (Catalog #A11055), donkey anti‐mouse 568 (Catalog #A10037), donkey anti‐rabbit 568 (Catalog #A10042), goat anti‐rabbit 488 (Catalog #A11008), goat anti‐rat 568 (Catalog #A11077) and biotinylated rabbit anti‐sheep (1:500, Vector Labs; Catalog #BA‐6000) which was additionally incubated for another 1 h 15 min with streptavidin Alexa Fluor 488 conjugate (1:500, Invitrogen; Catalog #S32354). Finally, sections were then mounted and coverslipped with DABCO (Sigma‐Aldrich; Catalog #D2522). Fluorescent images were obtained with a Leica SP8 laser scanning confocal microscope with LAS X software or with the Olympus VS120 slide scanner.

X‐34 (Sigma‐Aldrich; Catalog #SML1954) staining was performed as previously described (Ikonomovic et al. 2006). In brief, after incubation with primary and secondary antibodies, free‐floating sections were incubated for 10 min in a solution with X‐34 (100 μM). Images were obtained using a SP8 Leica confocal microscope as one stack per section with a 63× oil objective and zoom factor #1 with a Z stack of 20 μm and 1 μm distance for each confocal plane. For Thioflavin‐S (Thio‐S; Sigma‐Aldrich; Catalog #230456) staining, sections were stained with 0.02% Thio‐S after incubation with the corresponding antibodies. For DAPI staining, sections were incubated in a solution of DAPI‐PBS (4 μg/μL) for 30 s.

For Periodic Acid Schiff (PAS; Panreac, Barcelona, Spain; Catalog #132320) staining, sections were immersed in 0.5% periodic acid for 15 min and then incubated in Schiff's Reagent (Panreac; Catalog #171588) for 5 min, followed by three 5‐min washes in 0.4% sodium metabisulfite (Panreac; Catalog #131698). Sections were examined under a Nikon Eclipse 80i microscope, and images were acquired with a Nikon DS‐5 M high resolution digital camera using the ACT‐2 U imaging software (Nikon Corporation, Minato, Tokyo, Japan).

### 2.12 Image Analysis

Aβ, AT8, CD45, CD68, GFAP, MC1, PHF1, and P2RY12 loads were determined by calculating the hippocampal area occupied by immunopositive staining using the image analysis software ImageJ (v1.53c). Similarly, APP load was calculated, but in this case only the signal associated with Thio‐S‐positive plaques was considered for the quantification.

Plaque‐associated microglial loading (*n* = 15–20 plaques per mouse) was measured by defining three concentric circles with a distance from the plaque of 10, 20, and 30 μm respectively (Figure 4A). Images of the plaques were taken randomly within the hippocampal region and avoiding close plaques so as not to obtain overlapping data. Then, Iba1 and TREM2 loads were determined by dividing the area occupied by positive immunostaining by the area of each circle, using ImageJ (v1.53c).

PAS staining quantification was assessed by considering the number of clusters of PAS‐positive granules in the hippocampus. Additionally, the number of PAS‐positive granules in each cluster was counted, as well as the area occupied by them using the software ImageJ (v1.53c).

Seeding potential ratio of each brain homogenate was estimated by dividing the induced Aβ or tau hippocampal pathology load by the total biochemical amount of Aβ or tau in the respective brain extracts determined by MSD or ELISA, respectively.

### 2.13 Statistical Analysis

Statistical analysis was conducted using GraphPad Prism (9.4.1 version) software. All values are expressed as means ± SEM, with *p* ≤ 0.05 considered statistically significant. Data sets were tested for normality using the Shapiro–Wilk test. When normal distribution was confirmed, data sets were assessed with Student's *t* test for comparing two groups, and 1‐ or 2‐way ANOVA followed by Tukey's test for analyzing more than two. When data sets did not follow a normal distribution, the Kruskal–Wallis test was performed (comparison of more than two groups) followed by Dunn's post hoc test. Asterisks were assigned as follows: *p ≤ 0.05, **p < 0.01, and ***p < 0.001, and the number of biological replicates is expressed as “N”.

## 3 Results

### 3.1 Human AD and Aged 3xTg‐AD Brain Seeds Differentially Induce Amyloid Plaques Formation

As previously mentioned, propagation and spreading of misfolded proteins can be influenced by multiple factors such as the host, route of administration, strain identity, and time selected to allow the inoculum to propagate through the brain. Here, we employed brain homogenates from two different sources, 25 month‐old 3xTg‐AD mice (old‐Tg) displaying profound amyloid and tau pathologies, and a *postmortem* AD patient. These inocula were selected to determine which sample has more potential to induce and spread the amyloid pathology in the 3xTg‐AD model (Figures S1 and S2). First, we compared and characterized the Aβ content in these extracts (Figure S1B–H). Total Aβ (including Aβ38, Aβ40 and Aβ42) (Figure S1Bb1) and Aβ42 (Figure S1Bb2) levels showed a higher amount in old‐Tg samples compared to the AD brain extract (Figure S1B). Moreover, dot‐blot for 6E10, OC, and A11 antibodies showed elevated levels of misfolded proteinaceous species in the old‐Tg sample compared to the human brain (Figure S1C,D). Similarly, western‐blot analysis of the brain extracts revealed the presence of low molecular weight oligomers (dimers and pentamers) in aged 3xTg‐AD mice samples compared to the human sample (Figure S1E). A protein misfolding cyclic amplification (Aβ‐PMCA) assay showed the presence of seeding‐competent aggregates in the brain of both human and old‐Tg samples. The acceleration in aggregation promoted by the human AD sample was higher than that induced by the old‐Tg mice sample, suggesting that the human brain extract contains more competent seeds than the old‐Tg mice (Figure S1F,G). In addition, mass spectrometry analysis showed minor differences in Aβ fragments between the human and old‐Tg samples (Figure S1H). Next, histological characterization of the amyloid pathology exhibited an elevated number of plaques positive for 6E10, Aβ42 and Aβ40 antibodies in both human brain samples and old‐Tg mice (Figure S2A), indicating that both types of samples contain a significant amount of amyloid aggregates. Fibrillar aggregates were also stained with Thio‐S and could be observed in both types of samples, notably being remarkably higher the amount of Thio‐S aggregates in old‐Tg brain (Figure S2B). In addition, tau pathology was also assessed, confirming the presence of hyperphosphorylated and conformational tau aggregates in both sample types (Figure S2C). Overall, the data indicate that higher levels of soluble Aβ species were present in the aged transgenic mice compared to the human extract; however, more amyloidogenic potential was observed in the human AD sample as shown by the in vitro assay.

To test the amyloidogenic capacity of human and mice Aβ seeds in vivo, 3xTg‐AD mice were inoculated in the right hippocampus with human AD or old‐Tg mice brain homogenates and then aged for 10 months (Figure 1A,B). First, 6E10 (Figure 1C) and OC (Figure 1E) antibodies were used to evaluate the amyloid load in the hippocampus of these injected mice. Interestingly, the results showed that 3xTg‐AD mice that were inoculated with the human AD brain extract contained a higher amount of amyloid deposits compared to 3xTg‐AD mice that received brain extract from old‐Tg mice (Figure 1C–H), despite the inoculated samples from the AD patient displaying a lower level of Aβ compared to old‐Tg mice (Figure S1B). Notably, the data obtained with the 6E10 marker showed that the amyloid load levels were elevated in both ipsilateral and contralateral sides of 3xTg‐AD mice hippocampi inoculated with the human brain extract, compared to those that received PBS (increase of 74% ± 7.03% in between the ipsilateral sides and of 72% ± 7.47% with respect to the human contralateral side) and those that received brain extract from old‐Tg mice (increase of 62% ± 13.09% of the Aβ load in the ipsilateral sides) (one‐way ANOVA, *F*(4, 25) = 6.200, *p* = 0.0013, Tukey's multiple comparisons tests, Figure 1C,D). Likewise, a similar pathological pattern was observed with the OC marker, in which, the ipsilateral side of 3xTg‐AD mice treated with the human extract showed the highest amyloid load burden compared with the 3xTg‐AD mice treated with PBS (increase of 86% ± 1.49% with respect to the human ipsilateral side and of 77 ± 2.48% to the human contralateral side) and treated with the old‐Tg mice brain extract (the increase compared with the mouse ipsilateral side is 39% ± 9.98% and compared with the mouse contralateral is 59% ± 7.76%) (one‐way ANOVA, *F*(4, 24) = 10.66, *p* < 0.0001, Tukey's multiple comparisons tests, Figure 1E,F).

The increment on Aβ pathology could be influenced by the amount of Aβ levels contained in the old‐Tg or human seed. To normalize the results, the seeding activity ratio was calculated (amyloid burden/concentration of Aβ levels measured by MSD) to evaluate the seeding potency of both brain extracts. Indeed, the data indicate that the human brain extract induced quite a higher amyloid burden in the ipsilateral side of 3xTg‐AD mice compared to the ipsilateral side treated with brain extracts from the old‐Tg mice sample (reduction of the seeding ratio by 79% ± 7.05%, unpaired *t*‐test, *t*(10) = 3.988, *p* = 0.0026, Figure 1G). Similarly, the seeding activity ratio for OC antibody indicated robust amyloid deposition in the ipsilateral side of 3xTg‐AD mice inoculated with the human extract compared to the old‐Tg homogenate (difference of 58% ± 9.61%, unpaired *t*‐test, *t*(10) = 3.558, *p* = 0.0052, Figure 1H). Overall, these findings suggest that the human AD sample contained more potent amyloid seeds than the brain extract from the familial AD transgenic model.

### 3.2 Human AD and Old 3xTg‐AD Seeds Differentially Affect Tau Pathology

**FIGURE 2:**
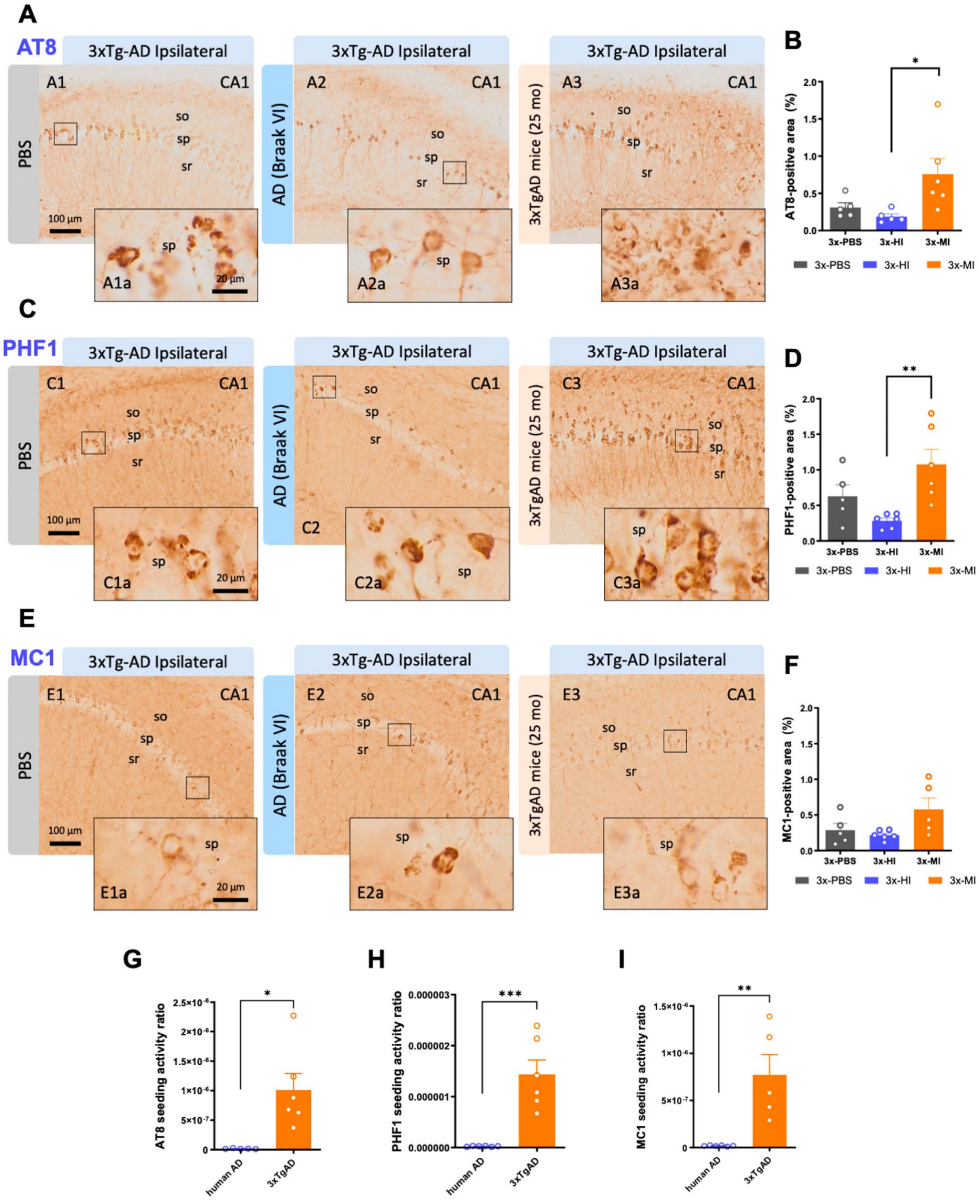
Human and transgenic mice AD seeds differentially accelerate tau pathology in 3xTg‐AD mice. (A) Hippocampal microscopic images immunolabeled for tau using the AT8 antibody in 3xTg‐AD mice treated in the ipsilateral side with PBS (A1, A1a), brain homogenates from an AD patient (A2, A2a), and with brain samples from an old 3xTg‐AD mice (A3, A3a). (B) AT8 positive area quantification in the ipsilateral side of the 3xTg‐AD treated with PBS, old‐Tg mice, and human brain homogenates. (C) Hippocampal microscopic images immunolabeled for hyperphosphorylated tau using the PHF1 antibody in 3xTg‐AD mice treated in the ipsilateral side with PBS (C1, C1a), brain homogenates from an AD patient (C2, C2a), and with brain samples from an old 3xTg‐AD mice (C3, C3a). (D) Quantification of the PHF1 positive area in the ipsilateral side of the 3xTg‐AD treated with PBS, old‐Tg mice, and human brain homogenates. (E) Hippocampal microscopic images immunolabeled for conformational hyperphosphorylated tau using the MC1 antibody in 3xTg‐AD mice treated in the ipsilateral side with PBS (E1, E1a), brain homogenates from an AD patient (E2, E2a), and with brain samples from an old 3xTg‐AD mice (E3, E3a). (F) Quantification of the MC1 positive area in the ipsilateral side of the 3xTg‐AD treated with PBS, old‐Tg mice, and human brain homogenates. (G–I) Activity ratio graphs corresponding to the ipsilateral side of 3xTg‐AD mice treated with human AD brain homogenate compared to old‐AD mice samples, according to the induced pathology immunolabeled with AT8 (G), PHF1 (H), and MC1 (I). The values represent means ± SEM. *N* = 5–6 per group. C, contralateral; H, human; I, ipsilateral; M, mouse; so, *Stratum oriens*; sp., *Stratum piramidale*; sr, *Stratum radiatum*. Scale bar: 100 μm (A1–A3, C1–C3, E1–E3) and 20 μm (A1a–A3a, C1a–C3a, E1a–E3a).

Tau aggregates can also act as prion‐like seeds, causing the misfolding and aggregation of normally folded tau (Frey et al. 2023; Holmes et al. 2014). Here, we also tested the capacity of the human and old‐Tg samples to develop tau pathology in 3xTg‐AD mice (Figure 2A). The AT8 and PHF1 antibodies were used to determine the accumulation of phospho‐tau pathology in the hippocampi of 3xTg‐AD mice. Also, the MC1 antibody was employed for the loading of conformational pathological tau in this region. The data showed that mice inoculated with the old‐Tg mice brain extract developed more phospho‐tau pathology compared to 3xTg‐AD mice treated with the human AD brain extract according to the results with both the AT8 antibody (increase of 74% ± 4.27% of the tau load, one‐way ANOVA, *F*(2, 13) = 4.722, *p* = 0.0287, Tukey's multiple comparisons tests, Figure 2A,B) and PHF1 antibody (increase of 74% ± 3.76% of the tau load, one‐way ANOVA, *F*(2, 14) = 6.944, *p* = 0.0080, Tukey's multiple comparisons tests, Figure 2C,D). Consistently, it was observed in the MC1 loading a higher trend in those 3xTg‐AD mice injected with old‐Tg mice brain extracts than with human AD or PBS (variation of 63% ± 4.71%, one‐way ANOVA, *F*(2, 13) = 3.549, *p* = 0.0589, Tukey's multiple comparisons tests, Figure 2E,F). In summary, these findings suggest that the old‐Tg mice contain more potent tau seeds than the human AD sample.

However, it was possible that the amount of tau levels contained in the old‐Tg or human extract could influence the increase in tau pathology observed in the injected brains. To normalize the results, the seeding activity ratio was calculated (tau burden/concentration of tau levels measured by ELISA) to evaluate the seeding potency of both brain extracts. Conversely to what is observed for the amyloid seeding activity ratio, these results showed that old‐Tg mice homogenates were able to seed a higher tau pathology in the ipsilateral side of 3xTg‐AD mice than the human AD samples, as calculated for AT8 (62% ± 17.26%, unpaired *t*‐test, *t*(9) = 3.238, *p* = 0.0102, Figure 2G), PHF1 (58% ± 11.68%, unpaired *t*‐test, *t*(10) = 5.008, *p* = 0.0005, Figure 2H) and MC1 (34% ± 11.62%, unpaired *t*‐test, *t*(9) = 3.878, *p* = 0.0037, Figure 2I). These findings suggest that the brain extract from the aged familial AD model 3xTg‐AD beared more potent tau seeds that the human AD sample.

### 3.3 Old 3xTg‐AD Seeds Impair Microglia Associated‐Plaque Response

**FIGURE 3:**
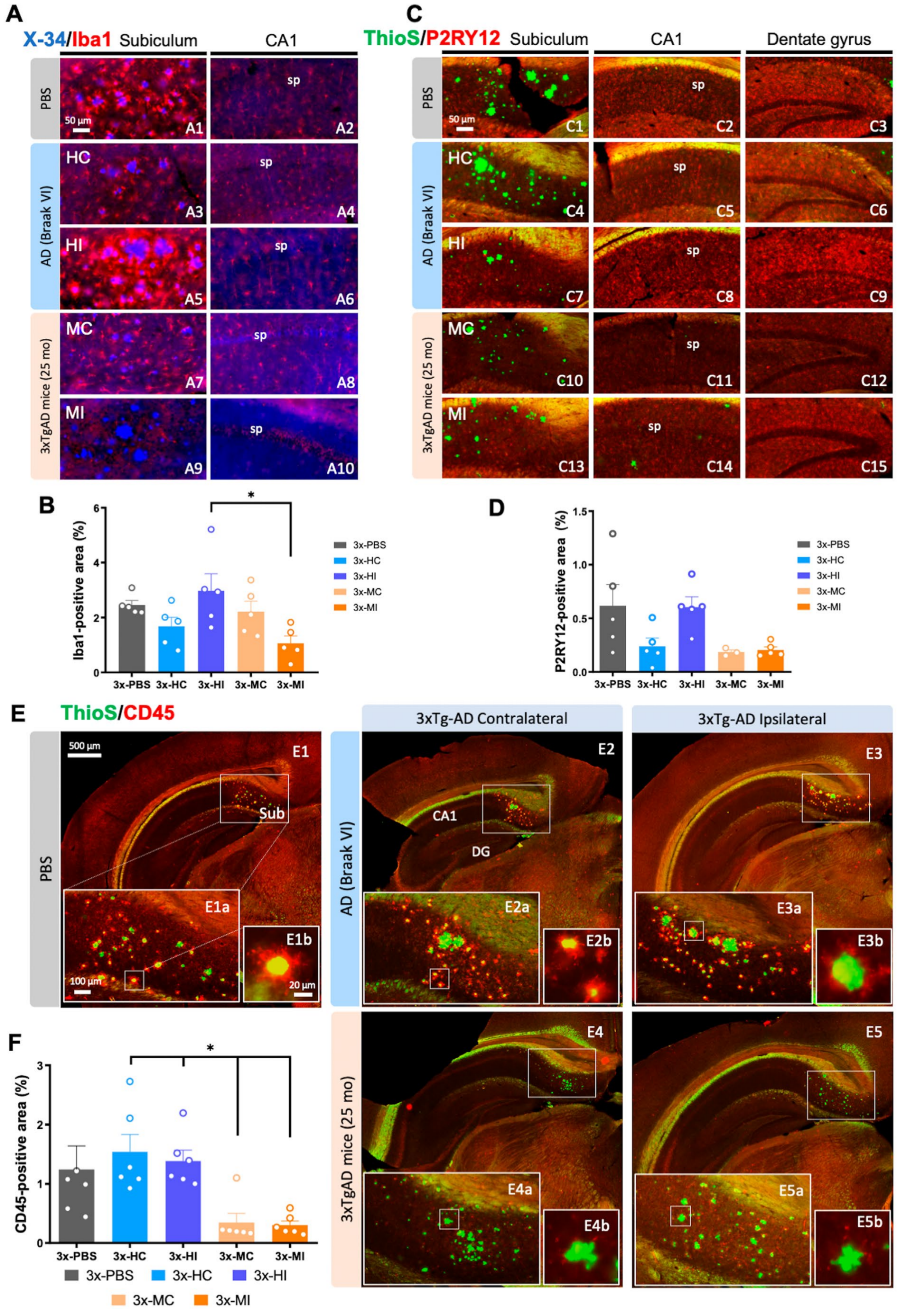
Human and transgenic mice seeds differentially affect the formation of plaque‐associated microglia in 3xTg‐AD mice. (A) Fluorescence microscopy images stained with X‐34 and Iba1 in the subiculum (A1, A3, A5, A7, and A9) and CA1 (A2, A4, A6, A8, and A10) hippocampal area. (B) Quantification of Iba1 positive area in 3xTg‐AD mice treated with PBS, human seeds, and old‐Tg seeds in both ipsilateral and contralateral side. (C) Fluorescence microscopy images stained with Thioflavin‐S and using the P2RY12 antibody in the subiculum (C1, C4, C7, C10, and C13), CA1 (C2, C5, C8, C11, and C14) and dentate gyrus (C3, C6, C9, C12, and C15). (D) Quantification of hippocampal P2RY12 positive area in 3xTg‐AD mice treated with PBS, human seeds, and old‐Tg seeds in both ipsilateral and contralateral sides. (E) Hippocampal fluorescence microscopy images stained with Thioflavin‐S and immunolabeled for CD45 in 3xTg‐AD mice treated with PBS (E1) and with brain homogenates from AD patient in the contralateral (E2) and ipsilateral (E3) side, and from old‐Tg mice in the contralateral (E4) and ipsilateral (E5) side. (F) Quantification of the CD45 load in PBS, human seeds treated mice and mice treated with the old‐Tg seeds in both ipsilateral and contralateral side. C, contralateral; H, human; I, ipsilateral; M, mouse; sp., *Stratum piramidale*; Sub, *Subiculum*. The values represent means ± SEM. *N* = 5–6 per group. Scale bar: 500 μm (E1–E5), 100 μm (E1a–E5a), 50 μm (A1–A10, C1–C15), 20 μm (E1b–E5b).

**FIGURE 4:**
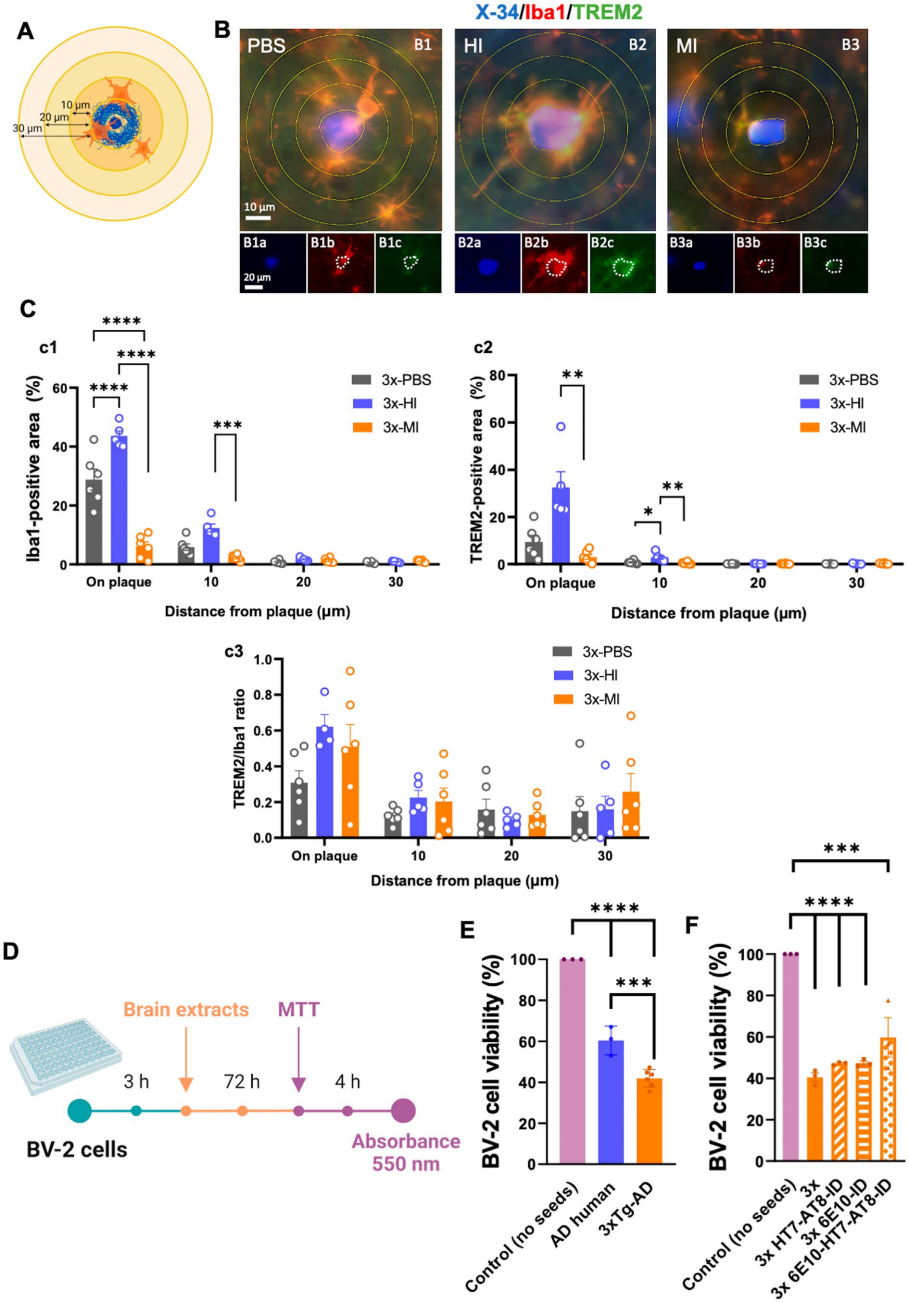
Iba‐1/TREM2 periplaque microglia were barely present in brain sections treated with old‐Tg sample. (A) Schematic representation of the regionalization of the plaque‐associated area for quantification. Three concentric circles were drawn outwards the outline of the plaque, with a radius of 10, 20, and 30 μm each. Then, microglia on plaque was assessed, as well as microglia at a distance of 10, 20, and 30 μm. (B) Fluorescence microscopy images stained with X‐34 and immunolabeled for Iba1 and TREM2 in 3xTg‐AD mice treated with PBS (B1) and with brain homogenates from AD patient (B2) and old‐Tg mice (B3) in the ipsilateral side. (C) Quantification of Iba1 load (c1), TREM2 load (c2), and TREM2/Iba1 ratio (c3). (D) Schematic timeline of the MTT assay. BV‐2 cells were seeded into a 96‐well plate and, after 3 h, brain extracts were added to the cells at a final concentration of 0.1 μg/μL. After incubation for 72 h, MTT was added to each well and further incubated for 4 h. Then, acid isopropanol was added and absorbance at 550 nm was measured. (E) BV‐2 cells treated with human, old‐Tg samples (0.1 μg/μL), and the control sample with no seeds. (F) BV‐2 cells treated with old‐Tg immunodepleted samples for Aβ, tau, and both Aβ and tau. H, human; I, ipsilateral; ID, immunodepleted; M, mouse. The values represent means ± SEM. *N* = 4–6 per group (C), *N* = 3–6 per group (E, F). Scale bar: 10 μm (B1–B3), 20 μm (B1a–B1c, B2a–B2c, B3a–B3c).

Neuroinflammation is a major hallmark in the neuropathology of AD, and microglial activation reflects the presence of brain damage (Dincgez Cakmak et al. 2019). In addition, recent evidence suggests that microglia take part in several pathogenic processes, such as amyloid and tau propagation, aggregation, plaque compaction, as well as protection against dystrophic neurites (Condello et al. 2015; Tran et al. 2023; Y. Wang et al. 2016). With this regard, we investigated the microglial dynamic toward amyloid plaques in 3xTg‐AD mice inoculated with human or with old‐Tg mice seeds, since the microglial response could be related to the differences found in the amyloid and tau loads in the inoculated 3xTg‐AD mice. Thus, we performed several immunohistological analyses with CD45, CD68, Iba1, P2RY12, and TREM2 antibodies to determine any changes in microglial load, state of activation, and distribution around the plaques and through the hippocampus (Figures 3, 4 and Figure S3). Iba1 load was significantly disminished (52% ± 10.99% reduction) in the ipsilateral side of the 3xTg‐AD hippocampus injected with the old‐Tg mice brain homogenate in comparison with mice inoculated with the human brain extract (one‐way ANOVA, *F*(4, 20) = 3.673, *p* = 0.0212, Tukey's multiple comparisons tests, Figure 3A,B). Similarly, loading of the homeostatic microglia marker P2RY12 showed a tendency to decrease in the brains injected with old‐Tg mice extracts in comparison with the human AD injected (variation of the 62% ± 4.67% in the ipsilateral sides, Figure 3C,D). Further investigating the microglia state in these injected brains, it was interesting that 3xTg‐AD mice injected with the old‐Tg mouse brain extract barely exhibited microglial clustering around plaques compared with mice injected with human brain homogenate or PBS (Figure 3E). Quantitative analysis of the hippocampal area occupied by the CD45‐positive immunostain revealed that 3xTg‐AD mice injected with the old‐Tg mice brain homogenate had significantly less CD45‐microglia in the hippocampal region in both the ipsilateral (78% ± 5.00% reduction) and contralateral (75% ± 10.90% reduction) sides in comparison with 3xTg‐AD mice treated with the human brain extract (one‐way ANOVA, *F*(4, 25) = 5.766, *p* = 0.0020, Tukey's multiple comparisons tests, Figure 3F). These results agree with those observed for the phagocytic microglia marker CD68 (Figure S3A). The quantitative analysis of the hippocampal area immunopositive for CD68 showed that 3xTg‐AD mice injected with the old‐Tg mice brain extract had significantly less microglia positive for CD68 in the ipsilateral (57% ± 8.52% reduction) as well as in the contralateral (48% ± 13.46% reduction) side when compared with 3xTg‐AD mice treated with the human AD inocula (one‐way ANOVA, *F*(4, 25) = 4.219, *p* = 0.0096, Tukey's multiple comparisons tests, Figure S3B).

Then, we examined the distribution and activation of microglia at different distances from the plaques using triple X‐34/Iba1/TREM2 labeled sections (Figure 4A–C). We measured the loading of total microglia (Iba1‐positive) and activated microglia (TREM2‐positive) at direct proximity with the plaque core and at 10, 20, and 30 μm away from the plaque core (Figure 4A). As shown in Figure 4B, Iba1‐microglia were abundant in close contact with plaques in 3xTg‐AD mice injected with PBS (Figure 4B1) or the human Aβ seeds (Figure 4B2); however, in those mice inoculated with old‐Tg mice seeds, periplaque microglia were almost absent (Figure 4B3). TREM2‐microglia were mostly present in direct contact with plaque cores from mice inoculated with the human seeds (see Figure 4B2c compared with B1c and B3c). The quantitative image analysis showed a significant increment of the microglial load that was in direct contact (Iba1: 86% ± 3.62%, TREM‐2: 91% ± 3.26%) or at 10 μm away (Iba1: 81% ± 3.30%, TREM‐2: 85% ± 6.53%) from the plaque core in 3xTg‐AD mice injected with human AD seeds compared to the groups injected with old‐Tg mice seeds, likewise with the PBS group (Iba1: 34% ± 8.18%, TREM‐2:71% ± 8.33% on plaque, and Iba1: 52% ± 8.84%, TREM‐2: 98% ± 0.77% for 10 μm away) (for Iba1 loading: two‐way ANOVA; Interaction *F*(6, 56) = 41.26 *p* < 0.0001, Row factor *F*(3, 56) = 221.0 *p* < 0.0001, Column factor *F*(2, 56) = 73.20 *p* < 0.0001, Tukey's multiple comparisons tests; for TREM2 loading: Kruskal–Wallis test, for on plaque group *p* = 0.0002 for 10 μm group *p* = 0.0024, Dunn's multiple comparisons test, Figures 4B, Cc1 and Cc2). These results also indicate that microglial cells did not respond toward amyloid plaques in mice injected with the old‐Tg mice brain homogenate compared to the PBS treated group. Moreover, the activation state surrounding plaques between groups was estimated by calculating the TREM2/Iba1 ratio. Interestingly, the data showed that the 3xTg‐AD mice inoculated with human AD brain homogenate showed a higher ratio value compared to PBS or old‐Tg mice treated group (Figure 4Cc3). These data indicate that the amyloid deposits seeded by the human brain homogenate activated the microglia response around plaques compared to the other groups, and that the old‐Tg mice homogenate could seed toxic strains that, in the end, reduce the presence of microglia‐associated plaque in the 3xTg‐AD injected mice.

Taking into consideration these findings, we next assessed whether the old‐Tg brain homogenate was toxic for microglia. For this purpose, we measured cell viability using the MTT assay in BV‐2 cells, a murine microglial cell line (Figure 4D) exposed to the brain homogenates (0.1 μg/μL) during 3 h postplating the cells. The results revealed a significant decrease (one‐way ANOVA, *F*(2, 9) = 162, *p* < 0.0001, Tukey's multiple comparisons tests, Figure 4E) in BV‐2 cell viability when treated with the human (40% ± 2.85%) or old‐Tg mice (58% ± 1.18%) seeds. Interestingly, a more profound effect in microglia viability was observed in cells treated with the old‐Tg mice seeds (Figure 4E), suggesting that the murine Aβ/tau seeds contain more toxic misfolded proteins than the human extract. Thus, murine brain extracts immunodepleted for Aβ and tau (by using 6E10, AT8 and HT7 antibodies respectively) (Figure S4A,B) were used to treat BV‐2 cells in a new assay, and the data showed that both Aβ and tau seeds were highly toxic to the BV2 cells (Figure 4F). Interestingly, this effect was partially reverted when immunodepletion of Aβ and tau was simultaneously performed, and the brain extracts ablated for these two pathogenic peptides were less toxic to the BV2 cells (Figure 4F, Figure S4C). In addition, no differences in astrocytes were found between groups by assessing the expression of the glial fibrillary acidic protein (GFAP) marker (Figure S5A,B).

### 3.4 The Reduction of Plaque‐Associated Microglia Promotes Neuritic Pathology

**FIGURE 5:**
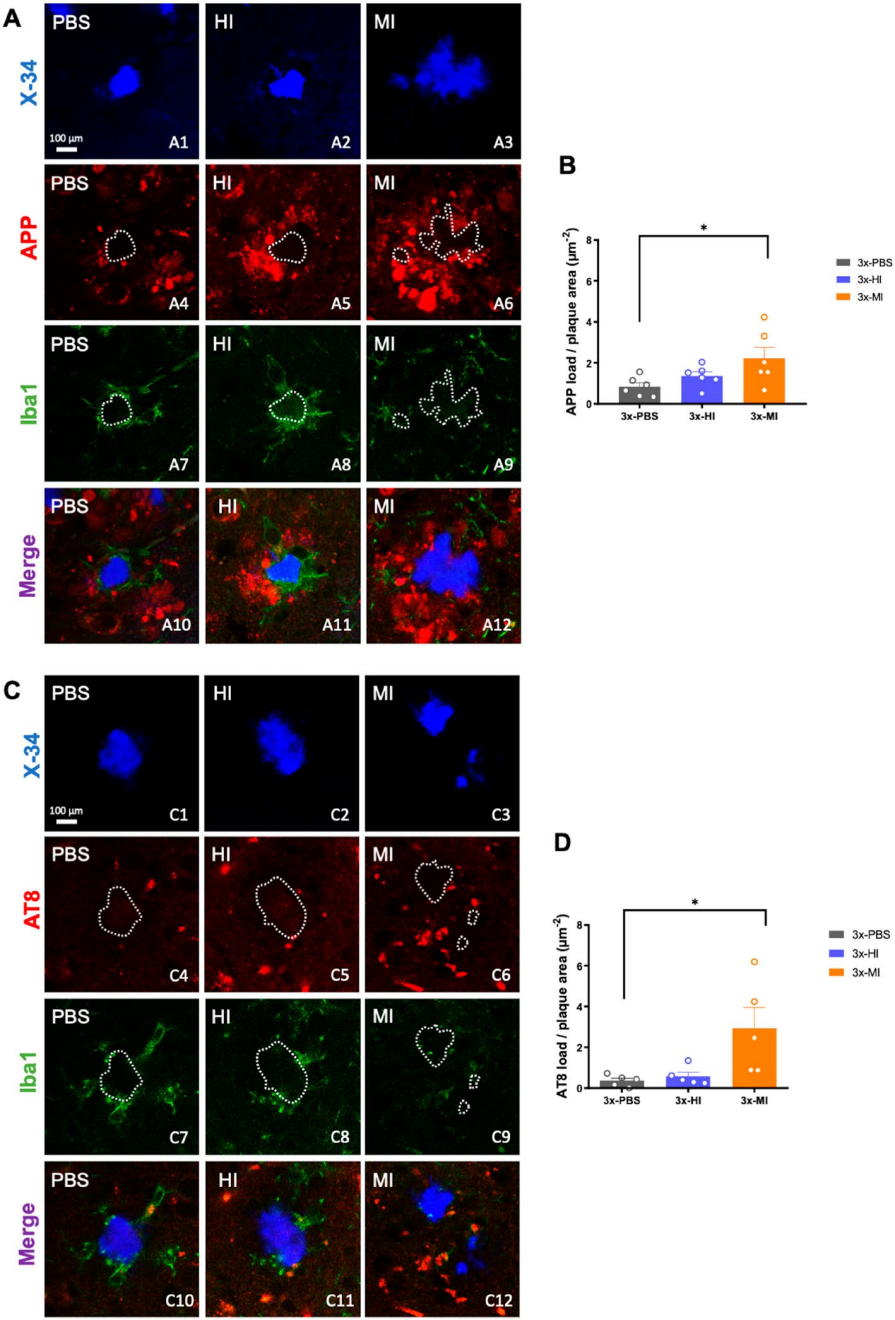
Increased dystrophic neurites pathology in 3xTg‐AD mice treated with seeds from old‐Tg mice. (A) Confocal images immunolabeled with APP and Iba1 microglia around X‐34‐positive amyloid aggregates in 3xTg‐AD treated with PBS (A1, A4, A7, A10), with brain homogenates from AD patient in the ipsilateral side (A2, A5, A8, A11) and treated with old‐Tg mice brain homogenates (A3, A6, A9, A12) in the ipsilateral side. (B) Quantification of APP positive dystrophic neurites divided by the plaque area in the hippocampus in 3xTg‐AD mice treated with PBS, human AD sample, and old‐Tg mice brain. (C) Confocal images of AT8 immunolabeled phospho‐tau along with Iba1 microglia and X‐34 positive amyloid plaques in the brains of the 3xTg‐AD mice treated with PBS (C1, C4, C7, C10), AD patient (C2, C5, C8, C11) and old‐Tg mice seeds (C3, C6, C9, C12) in the ipsilateral side. (D) Quantification of AT8 positive stain divided by the plaque area in the hippocampus of 3xTg‐AD mice treated with PBS, human AD sample, and old‐Tg mice seeds. H, human; I, ipsilateral, M, mouse. The values represent means ± SEM. *N* = 5–6 per group. Scale bar: 100 μm (A1–A12, C1–C12).

As previously reported, microglia‐uncovered plaque areas are associated with more severe axonal dystrophy (Condello et al. 2015). Taking into consideration our results, we sought to investigate whether the microglia‐absent plaques were associated with greater dystrophic neurite pathology. With this regard, we performed double immunolabeling for APP/Iba1 (Figure 5A), along with the labeling of amyloid fibrillar aggregates (X‐34). Quantitative analysis of the dystrophic neurite load was measured considering only the APP immunostain near the plaques, avoiding somatic APP signal. The data revealed a significant increment in the dystrophies associated with the amyloid plaques when inoculated with old‐Tg mice samples, in which microglia were not surrounding the plaques, compared to human (39% ± 9.38%) and PBS (62% ± 8.62%) groups (one‐way ANOVA, *F*(2, 15) = 4.044, *p* = 0.0394, Tukey's multiple comparisons tests, Figure 5B).

Furthermore, phosphorylated tau is known to accumulate within dystrophic neurites (Sanchez‐Varo et al. 2012; Trujillo‐Estrada et al. 2013). Thus, to corroborate the increment of neuritic pathology in microglia‐absent plaques in treated mice, we performed the colabelling for amyloid plaques (X‐34), microglia (Iba1) and phospho‐tau (AT8) (Figure 5C). Quantification of AT8 immunostaining associated with X‐34‐positive plaques revealed a significantly higher amount of phospho‐tau around the plaques in mice injected with old‐Tg mice samples compared to human (80% ± 6.30%) and PBS (87% ± 3.87%) groups (one‐way ANOVA, *F*(2, 12) = 5.511, *p* = 0.0201, Tukey's multiple comparisons tests, Figure 5D). Overall, these findings support previous evidence showing that the reduction or loss of the microglial barrier around the plaques is associated with more severe neuritic pathology.

### 3.5 Human Brain Seeds Do Not Accelerate Amyloid Pathology but Trigger the Formation of PAS‐Positive Aggregates in the hAβ‐KI Model

**FIGURE 6:**
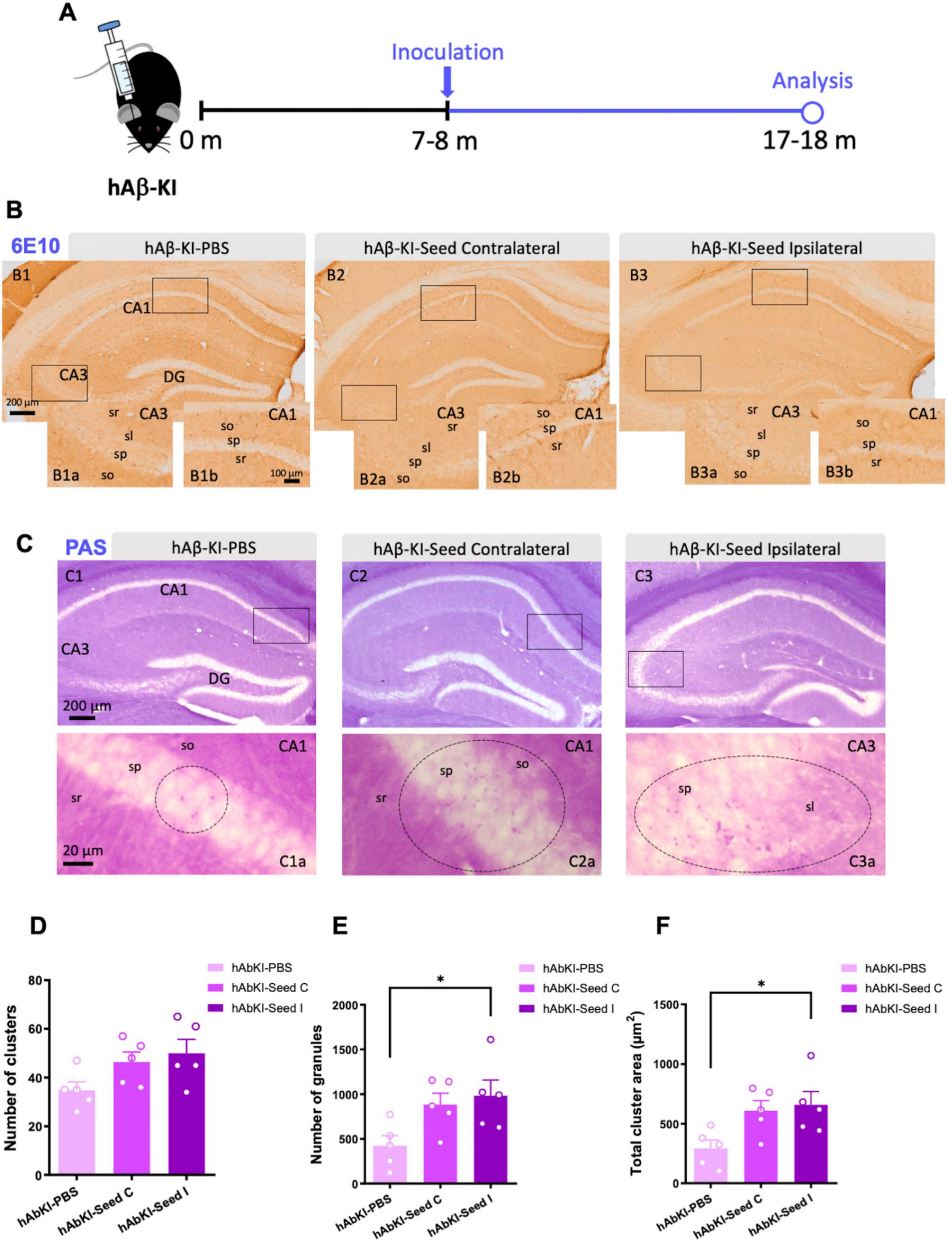
Human AD seeds do not accelerate amyloid pathology but stimulate the formation of PAS granules in hAβ‐KI mice. (A) Brain homogenates from AD patient were inoculated into the hippocampus of 7–8‐month‐old female hAβ‐KI mice, followed by a 10‐month incubation period. (B) Hippocampal microscopic images stained with 6E10 in hAβ‐KI mice treated with PBS (B1, B1a, B1b), with brain homogenates from AD patient in the contralateral (B2, B2a, B2b) and ipsilateral (B3, B3a, B3b) sides, showed no immunostaining with 6E10 antibody after 10 months of the injection. (C) Hippocampal microscopic images stained with PAS in hAβ‐KI mice treated with PBS (C1, C1a) and hAβ‐KI mice treated with brain homogenates from AD patient in the contralateral (C2, C2a) and ipsilateral (C3, C3a) side. (D–F) Quantification of the number of PAS clusters (D), total number of granules (E) and total area occupied of the granules (F) in the ipsilateral hAβ‐KI mice treated with human brain extract compared to hAβ‐KI mice treated with PBS. C, Contralateral; DG, Dentate gyrus; I, Ipsilateral; slu, *Stratum lucidum*; so, *Stratum oriens*; sp., *Stratum piramidale*; sr, *Stratum radiatum*. The values represent means ± SEM. *N* = 5 per group. Scale bar: 200 μm (B1–B3, C1–C3) and 100 μm (B1a–B3a, B1b–B3b, C1a–C3a, C1b–C3b).

Considering these previous findings, human brain extract contained more amyloidogenic seeds than the brain extracts from old‐Tg mice. Thus, we investigated the impact of the human AD seeds on amyloid aggregation in hAβ‐KI mice (Figure 6A). This novel transgenic line resembles a key model to closely mimic late‐onset sporadic AD, since no familial AD mutation is incorporated and it expresses wild‐type human Aβ sequence under murine physiological levels (Baglietto‐Vargas et al. 2021). Immunostaining with 6E10 antibody revealed no amyloid aggregation in the form of plaques after 10 months of the stereotaxic surgery (Figure 6B). Moreover, a longer time of incubation, 18 months, with seeds samples from cognitively normal and demented AD individuals showed that these brain extracts were not able to generate amyloid plaques when injected into hAβ‐KI mice hippocampus either (Figure S6). The seeding capacity of these inocula was evaluated in APP/PS1 mice and showed seeded pathology after 4 months of incubation (Figure S6).

Since the hAβ‐KI model develops age‐dependent accumulation of *corpora amylacea* aggregates (Baglietto‐Vargas et al. 2021), similar to other AD models and human AD brains (Auge et al. 2017; Manich et al. 2011; Riba et al. 2019; Wander et al. 2020, 2022), we investigated the impact of human seeds on this AD‐associated pathological feature. Our data showed (Figure 6D) that after 10 months of the stereotaxic injections, an increment in the number of Periodic Acid Schiff‐positive clusters was detected in hAβ‐KI mice that received human brain homogenates compared to PBS treated mice, including the ipsilateral (28% ± 6.03%) and contralateral side (22% ± 4.97%). In addition, the total number of granules (54% ± 9.73%) (one‐way ANOVA, *F*(2, 12) = 4.469, *p* = 0.0354, Tukey's multiple comparisons tests, *p < 0.05) and the area (54% ± 8.76%) (one‐way ANOVA, *F*(2, 12) = 4.753, *p* = 0.0302, Tukey's multiple comparisons tests, *p < 0.05) occupied by these granules in the cluster were significantly higher in the ipsilateral side of hAβ‐KI mice that received human brain homogenates compared to PBS treated mice (Figure 6E,F). Therefore, the administration of human competent seeds in hAβ‐KI mice is able to accelerate the formation of PAS aggregates despite the formation of plaques not being induced.

## 4 Discussion

Here, we demonstrated that native brain AD seeds from human and aged 3xTg‐AD mice have a distinct impact on the propagation of amyloid/tau pathology in the hippocampus of these transgenic mice. In addition, these different seeds induced pathology affecting the microglial viability and clustering around the plaques, leading to more severe neuritic pathology. Moreover, the human Aβ seeds were able to induce and propagate amyloid pathology more efficiently than those derived from aged 3xTg‐AD. However, these aggregates from human AD brain or transgenic mice were not able to induce amyloidosis in the brain of hAβ‐KI mice, indicating that the amount of amyloidogenic seeds, time of incubation, or multiple pathogenic insults might be needed to produce amyloid plaques in this model along its lifespan.

Aβ seeding mechanisms are influenced by several parameters, mainly including the source of the injected Aβ seed, the host, the administration route, and the incubation period (Eisele et al. 2010; Hamaguchi et al. 2012; Meyer‐Luehmann et al. 2006; Morales et al. 2012; Ulm et al. 2021; Walker 2018). Here, we determined the pathological potential of different brain extracts and their capacity to trigger the formation of Aβ plaques. The Aβ levels were higher in the murine brain extracts compared to the human AD specimen, which might explain, at least in part, their different amyloidogenic capacity to promote the formation of Aβ aggregates in a host. Surprisingly, the human AD extracts that exhibited lower Aβ levels than the murine extracts were most successful in inducing amyloid pathology as measured by amyloid burden and number of plaques. These results indicate that the human brain seeds had different amyloidogenic properties that may be influenced by the Aβ strains, facilitating the formation of amyloid aggregates as compared to the old‐Tg mice seeds. Indeed, novel findings in human brain tissue strongly suggest the presence of different Aβ strains across patients (Condello et al. 2018; Ghosh et al. 2021; Konstantoulea et al. 2022; Levine 3rd and Walker 2010; Qiang et al. 2017; Rasmussen et al. 2017; Walti et al. 2016), resulting in a distinctive and specific type of aggregates, pattern of propagation, immune responses, or neuritic damage, which may explain the phenotypic heterogeneity observed in human AD cases (Condello and Stoehr 2018; Duran‐Aniotz et al. 2021; Gomez‐Gutierrez et al. 2023; Li et al. 2022). Supporting this hypothesis, a recent study has also shown that posttranslational modifications or maturation of the amyloid peptide, such as N‐terminal truncated and pyroglutamate Aβ (Aβ_N3pE_) and phosphorylated Aβ (Aβp_Ser8_), are also a major factor driving the pattern and type of amyloid aggregates formed (Li et al. 2022). Thus, the presence of different Aβ conformations and oligomeric ensembles in the inoculum from human or transgenic AD samples may result in the different pattern and rate of propagation observed in our in vivo study using the 3xTg‐AD mice.

Moreover, several limitations may affect the propagation of the amyloid aggregates and should also be taken into consideration. For example, the differences in amyloid induction may also be influenced by the fragmentation of the Aβ seeds, since different conformations may have distinct susceptibility to mechanical disruption by extended sonication that increases the seeding capacity of the brain extract (Langer et al. 2011). In addition, further studies with other AD cases and brain regions are necessary, since AD individuals display different misfolded Aβ aggregates, which can significantly affect the propagation and formation of amyloid plaques in the brain in a different manner (Condello and Stoehr 2018; Duran‐Aniotz et al. 2021; Gomez‐Gutierrez et al. 2023; Li et al. 2022), and it is possible that different results may be obtained when comparing with the old‐Tg samples. Furthermore, it is important to consider the genetic risk polymorphisms containing each AD case, such as the *APOE* isoform, because it modulates the structure, composition, and progression of Aβ and tau aggregates, and it may explain the differences in amyloid and tau propagation between human and rodent (Xia et al. 2024).

In recent years, multiple studies have shown that microglial cells affect amyloid plaque formation, growth, and compaction (Baik et al. 2016; Casali et al. 2020; Condello et al. 2015; Sosna et al. 2018; Spangenberg et al. 2019). Once the Aβ fibrils accumulate in the brain parenchyma, the microglial cells sense changes in the extracellular matrix stiffness or roughness, attracting such cells through specific mechanical receptors to Aβ aggregates, and forming a physical barrier that avoids the outward extension of Aβ fibrils (Baik et al. 2016; Condello et al. 2015; Sosna et al. 2018; Spangenberg et al. 2019). Likewise, the microglial cells are also responsible for disseminating Aβ seeds from pathological to other unaffected brain areas due to their migrating and phagocytic capacity (d'Errico et al. 2022), showing that microglial cells have multiple roles in the context of amyloid pathogenesis. Taking into consideration these previous findings, we observed that microglia did not react by clustering tightly around the fibrillar aggregates in 3xTg‐AD mice treated with old‐Tg brain samples compared with the human AD extract, suggesting that the plaques templated by this inoculum in the 3xTg‐AD mice interact with or affect microglial tropism toward plaques.

In order to identify the potential mechanisms underlying this alteration in plaque‐associated microglia, we first determined the expression of TREM2 on the microglial cells associated with fibrillar plaques and in the near area. TREM2 is a relevant receptor that recognizes changes in the lipid microenvironment, as occurs in Aβ plaques (Gratuze et al. 2018). Recent studies in multiple animal models of AD showed that TREM2 deficiency affects the clustering of microglia around the plaques, making them more diffuse and surrounded by more neuritic damage, suggesting that the microglial response to plaques is dependent on TREM2 expression (Jay et al. 2015; Puntambekar et al. 2022; Takahashi et al. 2005; Ulrich et al. 2014; Wang et al. 2015; Wang et al. 2016; Wood et al. 2022). Here, we determined a lack of TREM2 expression in microglial cells surrounding the plaques, which can lead to the observed decrease in Aβ plaque compaction and more neuritic damage (Jay et al. 2015; Puntambekar et al. 2022; Takahashi et al. 2005; Ulrich et al. 2014; Wang et al. 2015; Wang et al. 2016; Wood et al. 2022). Along this line, the ratio of TREM2/Iba1‐positive cells was similar in the different conditions; however, this expression was reduced in 3xTg‐AD mice inoculated with old‐Tg seeds, suggesting that instead of a loss of function, a potential degenerative process of microglial cells might be occurring. Likewise, the amount of Iba1‐positive cells in 3xTg‐AD mice inoculated with old‐Tg seeds was significantly decreased at periplaque and interplaque areas, compared to the other experimental conditions. Whether this decline in Iba1‐cells represents cell death or loss of expression of this marker needs to be further evaluated. However, the fact that there is a significant loss of Iba1 cells is indicative of microglial abnormality that might compromise their survival. Further confirming this, an in vitro assay using BV‐2 cells determined that the murine brain extracts affected more significantly the viability of these cells compared to PBS and human brain groups. Further analysis with immunodepleted brain samples for Aβ and tau together showed that both seeds may contain toxic strains that affect the viability of the BV‐2 cells. In regard to these evidences, a previous study from our lab supports these results, in which it shows that soluble phospho‐tau strains from both AD cases and Thy‐tau22 hippocampi triggered microglia death (Sanchez‐Mejias et al. 2016). Likewise, old‐Tg brain accelerated the accumulation of phospho‐tau recognized by AT8 and PHF1 antibodies in 3xTg‐AD mice, and therefore, increased the amount of tau pathology that can modify the microglia vulnerability. Moreover, we cannot discard the possibility that Aβ plaques can also dysregulate the surrounded microglia, creating a stressful hypoxic environment that can affect the survival/activation of these cells by mitochondrial dysfunction (March‐Diaz et al. 2021). In addition, the microglia could be more vulnerable to the aging process when using murine Aβ seeds. With this regard, a new population of microglial cells named terminally inflammatory microglia (TIMs) has been described in AD cases and old Tg‐mice, and its frequency is influenced by *APOE4* and age (Millet et al. 2024). This population shows an inflammatory exhausted‐like state that results in an impaired capacity for Aβ clearance, and it is also possible that old‐Tg samples influence the formation of TIMs, reducing the capacity of the microglia cells to respond and cluster around the plaques. Nevertheless, further studies are necessary to determine this potential vulnerable microglia state when inoculating old‐Tg brain extracts versus human seeds. Overall, our data suggest that the inoculum from old‐Tg mice contains more toxic molecules than its human counterpart and affects the viability of the microglial cells or induces a toxic plaque‐associated environment that may lead to a vulnerable unresponsiveness microglia state. These effects seem to be specific to microglial cells, since no changes in cell density are observed in astrocytic cells.

Blocking microglial cells clustering around Aβ plaques induced by genetic or pharmacological approaches resulted in more neuritic damage and tau pathology (Condello et al. 2015; Gratuze et al. 2021; Gratuze et al. 2023; Lee et al. 2021; Leyns et al. 2019; Y. Wang et al. 2016). Indeed, our study supports this previous evidence, showing that the reduction of microglial cells surrounding the Aβ plaques when inoculating old‐Tg mice samples triggers the formation of dystrophic neurites. These data suggest that the old‐Tg mouse inoculum contained seeds able to induce the formation of more toxic Aβ/tau isoforms that affect the viability of the microglial cells and reduce the clustering of the Aβ aggregates, resulting in a significant increase in the neuritic damage. In addition, further studies such as behavioral tests are also necessary to support these findings and determine the impact of these seeds and neuritic pathology on cognition.

Besides the inoculum, other factors, such as the type of host model, are important in modulating the aggregation and propagation rate of misfolded proteins. As previously mentioned, many studies using murine lines of early‐onset Alzheimer's Disease (EOAD) have shown that both the incubation time and the route of administration are critical factors in the type of Aβ aggregates that are generated (Eisele et al. 2010; Hamaguchi et al. 2012; Meyer‐Luehmann et al. 2006; Morales et al. 2012; Ulm et al. 2021; Walker 2018). This kind of AD models bears mutations for familial genes of such disease, making them essential to understand the basis of amyloid pathology progression. Nevertheless, it is important to note that most of the seeding studies have been performed using murine models of EOAD (reviewed in (Ulm et al. 2021)), which represent the minority form of the AD human cases. Therefore, new animal models that more closely mimic the majority of cases (late‐onset Alzheimer's Disease cases; LOAD) have been developed in recent years, such as the hAβ‐KI line created by MODEL‐AD (Baglietto‐Vargas et al. 2021). This model is a knock‐in for human Aβ, in which the wild‐type human Aβ sequence has been inserted into the mouse *App* gene locus, meaning that the Aβ peptide expression is physiological, for being under the control of the endogenous mouse promoter, and lacks any mutation. This renders the hAβ‐KI a proper model to investigate the Aβ propagation in a model of LOAD.

Consistent with previous studies in APP models (reviewed in (Ulm et al. 2021)), in our study, the AD brain inoculum was able to induce amyloid pathology in 3xTg‐AD mice; specifically, both diffuse and neuritic plaques formation was accelerated. On the contrary, this inoculum was not able to induce the formation of amyloid plaques in hAβ‐KI mice, suggesting that the human homogenate accelerated the amyloid pathology in a model that is highly amyloidogenic and generates aggregates with age. However, the inoculum did not have the potential to trigger amyloid plaques during the lifespan of the LOAD line. Multiple reasons can be responsible for the absence of amyloid aggregates in the hAβ‐KI. These include: (i) the proteostasis machinery is sufficiently effective to remove the formed seeds in this model, (ii) a more potent amyloidogenic sample is necessary to generate plaques, or (iii) other pathogenic factors are necessary to complement the pathogenic potential of the inoculum (Brettschneider et al. 2015; Jucker and Walker 2013, 2018). In this regard, a previous study showed that they were able to induce Aβ deposition in a human wild type‐APP‐overexpressing model after inoculation with human AD brain extracts and incubation for 585 days (Morales et al. 2012), suggesting that amyloid aggregates formation may require a longer period of time in a KI model. For this reason, a longer time of incubation (18 months) from different demented and nondemented human samples containing amyloid pathology was also assessed. However, no aggregates were found despite the longer time employed. Thus, given the heterogeneity in human AD patients, it could be possible that the brain homogenates used bear different Aβ strains with distinct seeding properties that also depend on the region and tropism (Duran‐Aniotz et al. 2021). Therefore, future studies should aim to inoculate the hAβ‐KI model with seeds from AD patients with high amyloidogenic potential and also from different brain areas.

Furthermore, it is known that the hAβ‐KI mice accumulate PAS aggregates with aging, and the ablation of Aβ production reduces their formation, suggesting that Aβ triggers the accumulation of PAS granules (Baglietto‐Vargas et al. 2021). Thus, our study also supports this previous evidence and shows that hAβ‐KI mice inoculated for 10 months with brain extract from an AD patient displayed an increment in the formation and size of PAS granules. The PAS aggregates are composed by carbohydrate‐rich deposits from degenerating astrocytes, neurons, and oligodendrocytes (Manich et al. 2014; Wander et al. 2020), and they have been related to alteration of waste clearance processes from the brain parenchyma to the meninges and cervical lymph nodes (Riba et al. 2019). These aggregates have been detected in several animal models of accelerated aging and other neurodegenerative disorders, as well as in human brain tissue (Pisa et al. 2016; Riba et al. 2019, 2021; Wander et al. 2020, 2022). Thus, the acceleration of the formation of PAS granules could be indicative that neurodegenerative processes have been earlier induced in the hAβ‐KI mouse after the brain extract was inoculated.

## 5 Conclusions

Overall, this study shows that misfolded AD‐associated proteins from different sources modulate the formation of Aβ plaques and phospho‐tau at different rates, and shows that the human seeds analyzed contain a different pool of strains that accelerate the formation of amyloid aggregates when compared to the old‐Tg brain samples. Likewise, AD murine seeds trigger an unresponsiveness microglial state, which causes the loss of plaque microglial coverage and affects the formation of Aβ plaques. Moreover, a different amyloid propagation rate occurs in a familial rodent model of AD compared to a sporadic form, indicating that quite a longer time would be necessary, and/or multiple insults, to generate Aβ plaques in this late‐onset animal line.

The importance of this study relies on the fact that these data may lead to a better understanding of the differences between rodent and human amyloid strains, which could impact in a differential manner the development of the AD pathology and explain disease heterogeneity and clinical variants. Unraveling the differences in these pathogenic mechanisms is important to understand the intrinsic differences between animal models and human AD, so we can advance in the development of therapeutic strategies with the potential to delay the onset of this neurodegenerative disorder.

## Author Contributions

J.A.‐L., D.B.‐V. designed the work. J.A.‐L., C.N.‐D., K.D.H., M.M.T.N., F.J.C.‐M., S.Z., F.B., M.L.G., L.T.‐E., S.F., A.C.M., M.B.‐L., N.G. performed the histological and molecular studies. C.D.C. supervised and maintained the colony. M.L.G., F.B. performed the Mass Spectrometry analysis. C.C.‐M. and J.A.G.‐L. performed the in vitro cellular study. J.A.‐L., M.L.G., R.M., A.G., F.M.L., D.B.‐V. wrote the manuscript. All authors read and approved the final version of the manuscript.

## Ethics Statement

This study was performed in accordance with National Institutes of Health (NIH) and with the Spanish and the European Union regulations (RD53/2013 and 2010/63/EU) and approved by the Animal Research Committee from the University of California, Universidad de Malaga, and the University of Texas Health Science Center at Houston. Experiments and procedures with animals were designed to minimize animal suffering and reduce the number of animals used. The utilization of brain samples was approved by the corresponding ethics committees at the University of California, Irvine, and The University of Texas Health Science Center at Houston.

## Conflicts of Interest

The authors declare no conflicts of interest.

## Supporting information


Figure S1.

Figure S2.

Figure S3.

Figure S4.

Figure S5.

Figure S6.

## Data Availability

The datasets supporting the conclusions of this article are available in the RIUMA repository at the University of Malaga [https://doi.org/10.24310/riuma.28147].
