# Supplementary figures and images for: Human and Mouse Alzheimer's Seeds Differentially Affect Amyloid Deposition and Microglia‐Dependent Plaque Response in Aged Mice
Source: Aging Cell. 2025 May 13;24(8):e70094. doi: 10.1111/acel.70094 (PMC12341799; doi:10.1111/acel.70094)

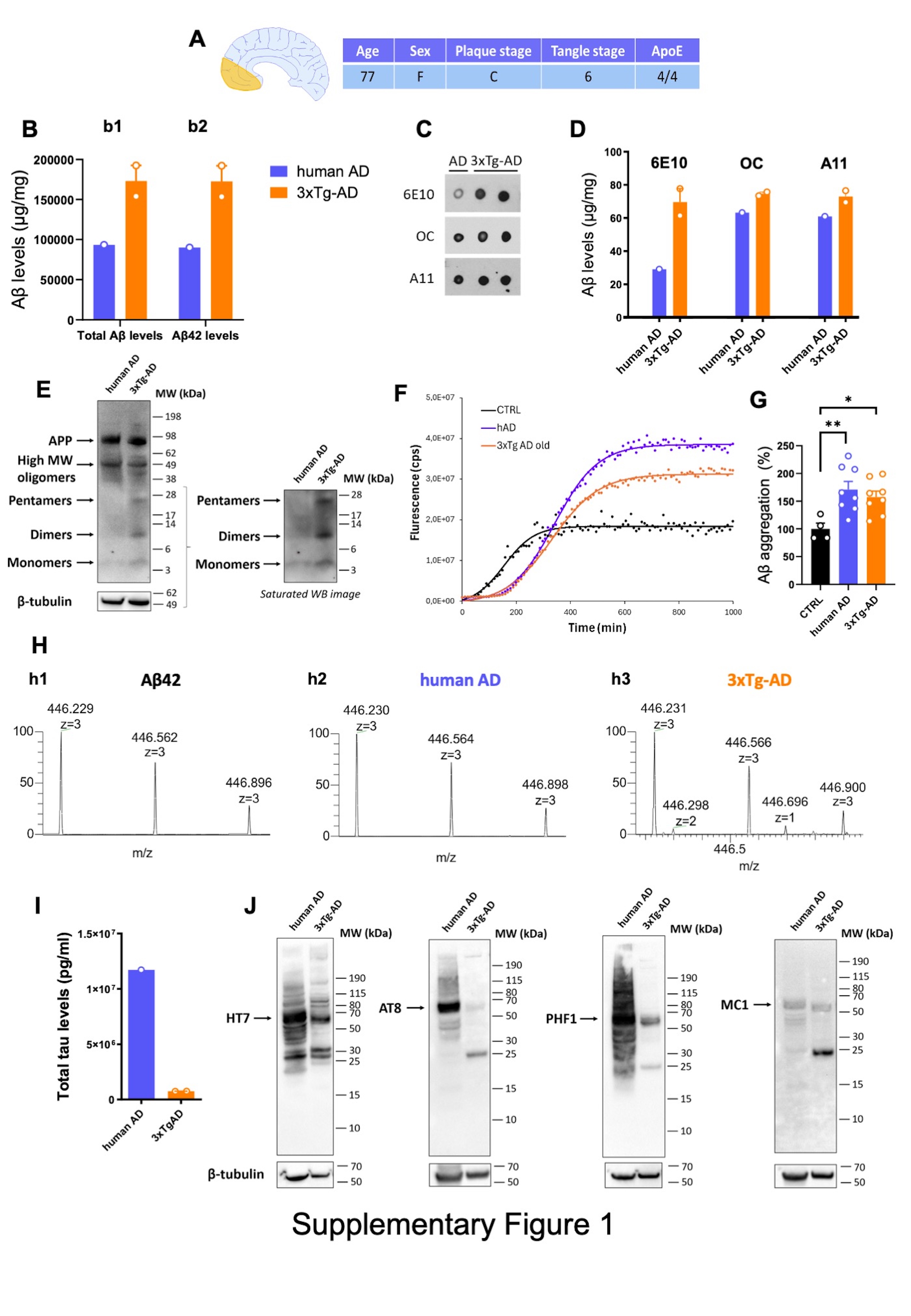

Supplement: Supplementary file 1 — Figure S1. [file ACEL-24-e70094-s004.jpeg]

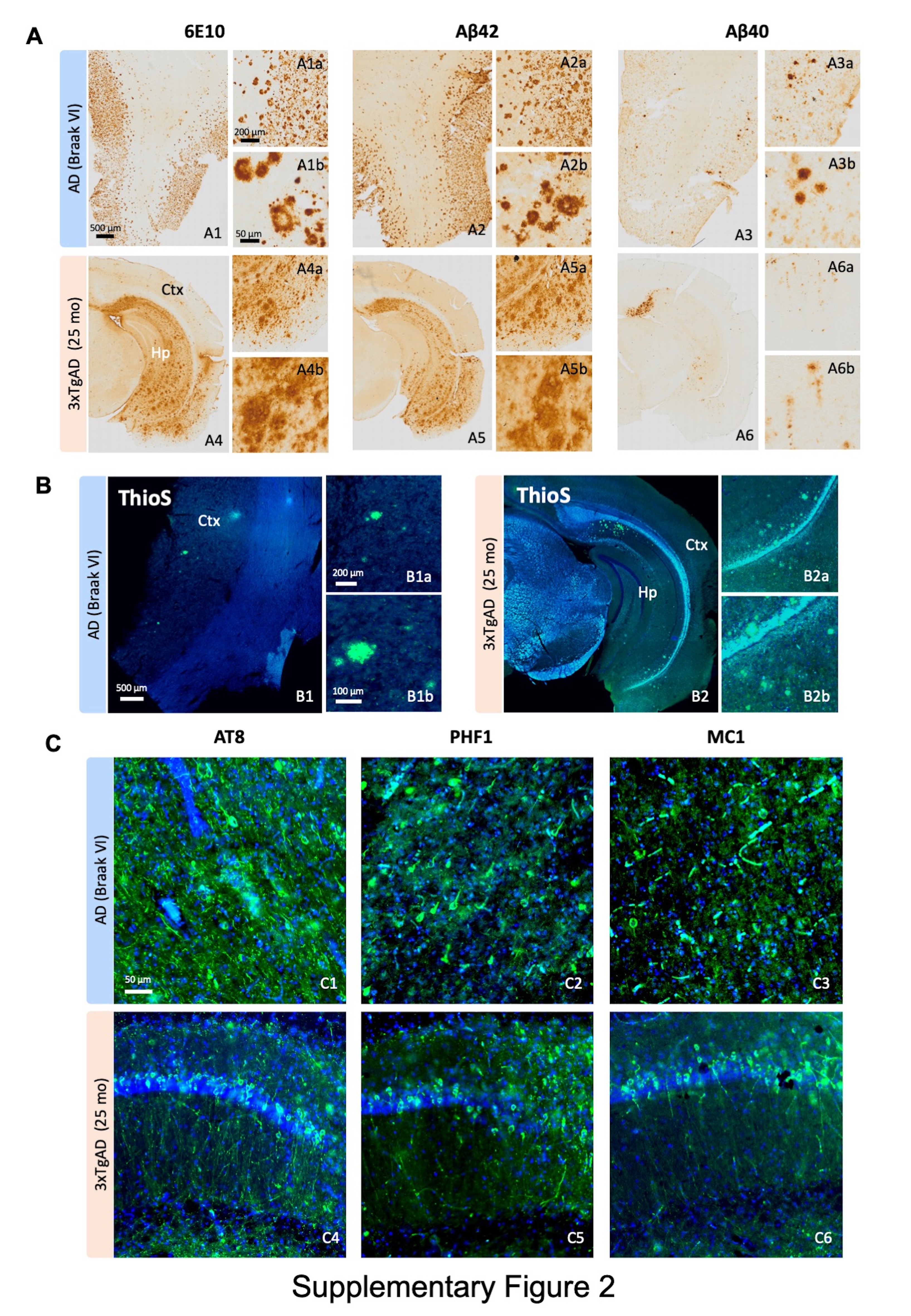

Supplement: Supplementary file 2 — Figure S2. [file ACEL-24-e70094-s001.jpeg]

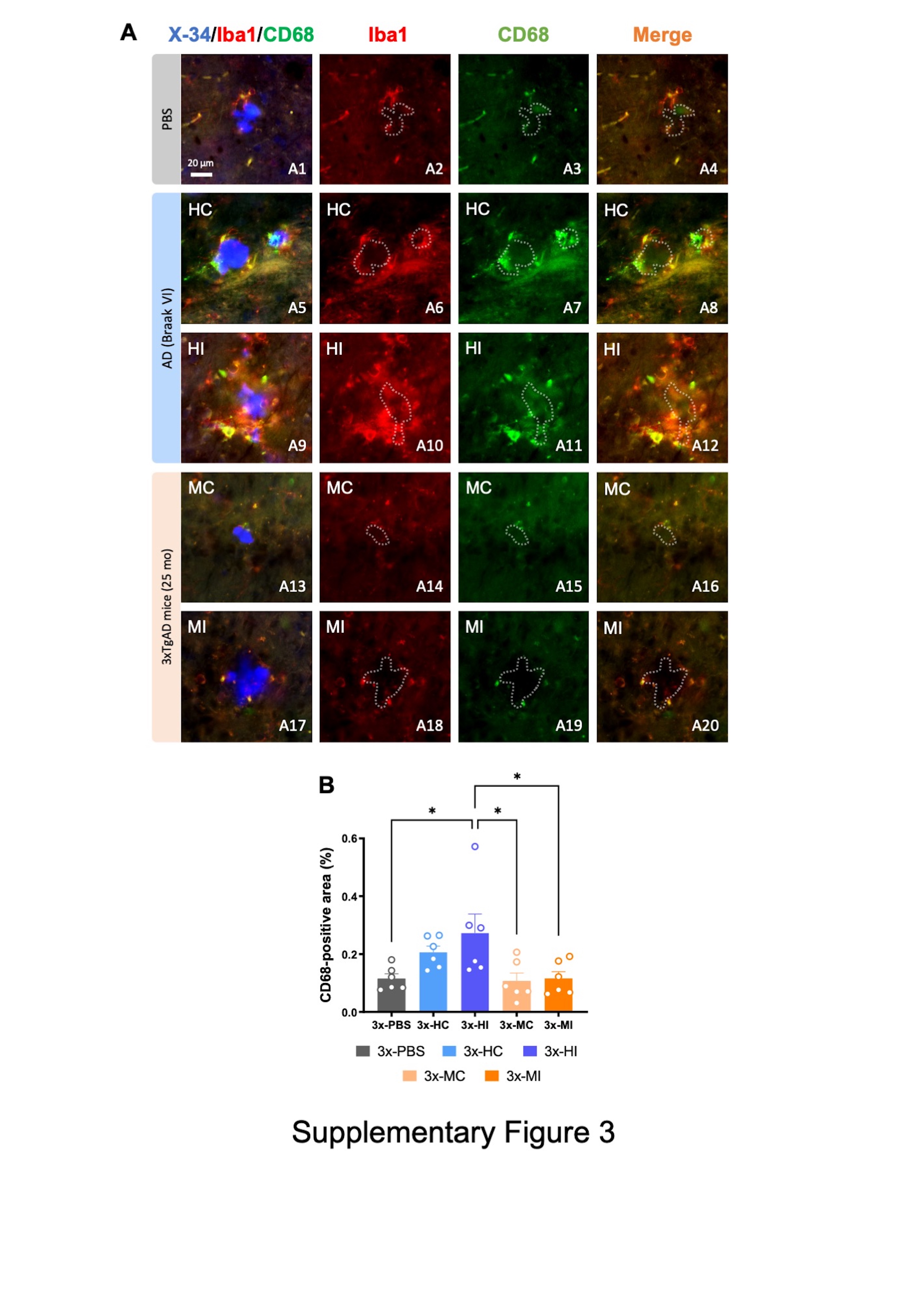

Supplement: Supplementary file 3 — Figure S3. [file ACEL-24-e70094-s003.jpeg]

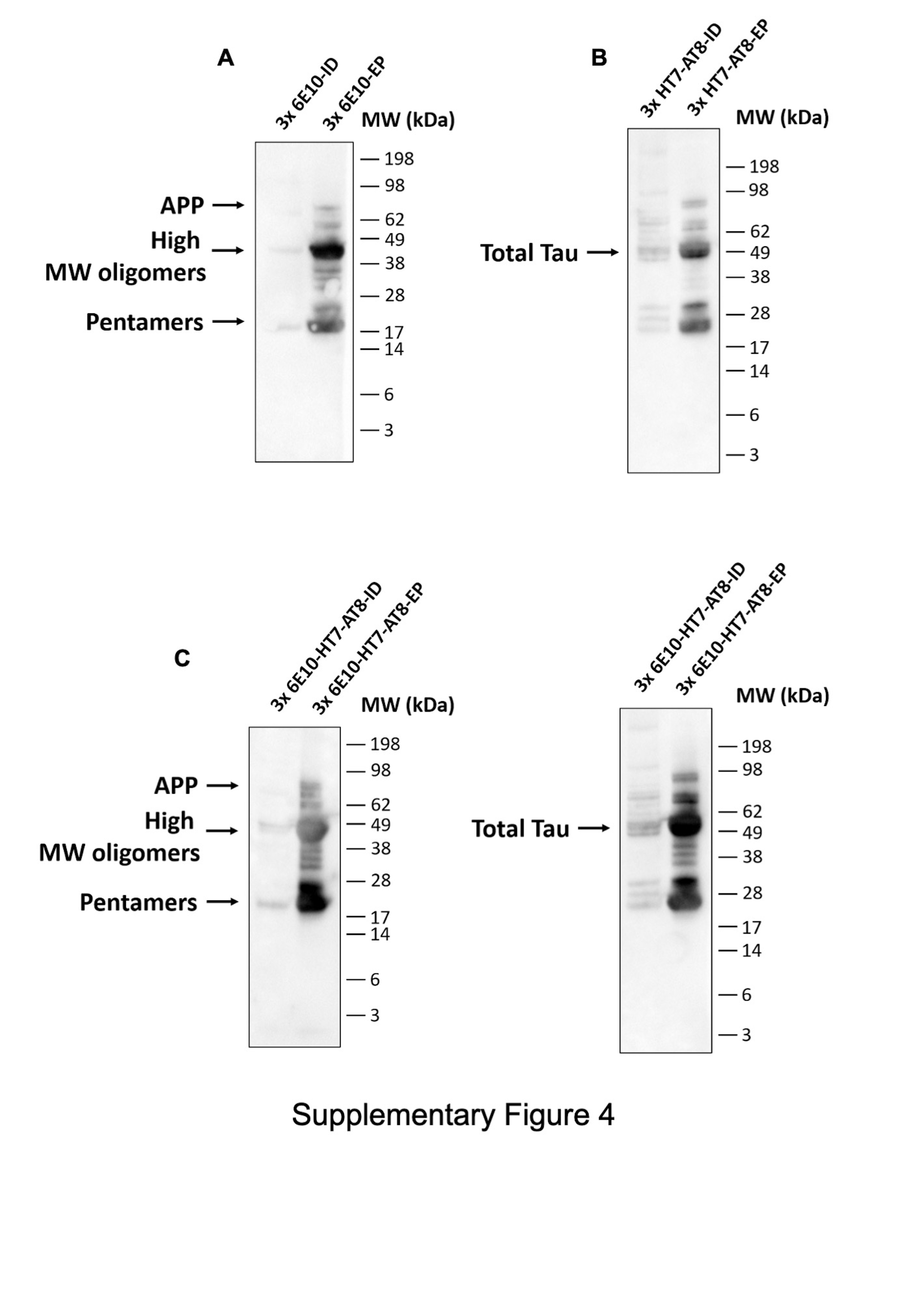

Supplement: Supplementary file 4 — Figure S4. [file ACEL-24-e70094-s006.jpeg]

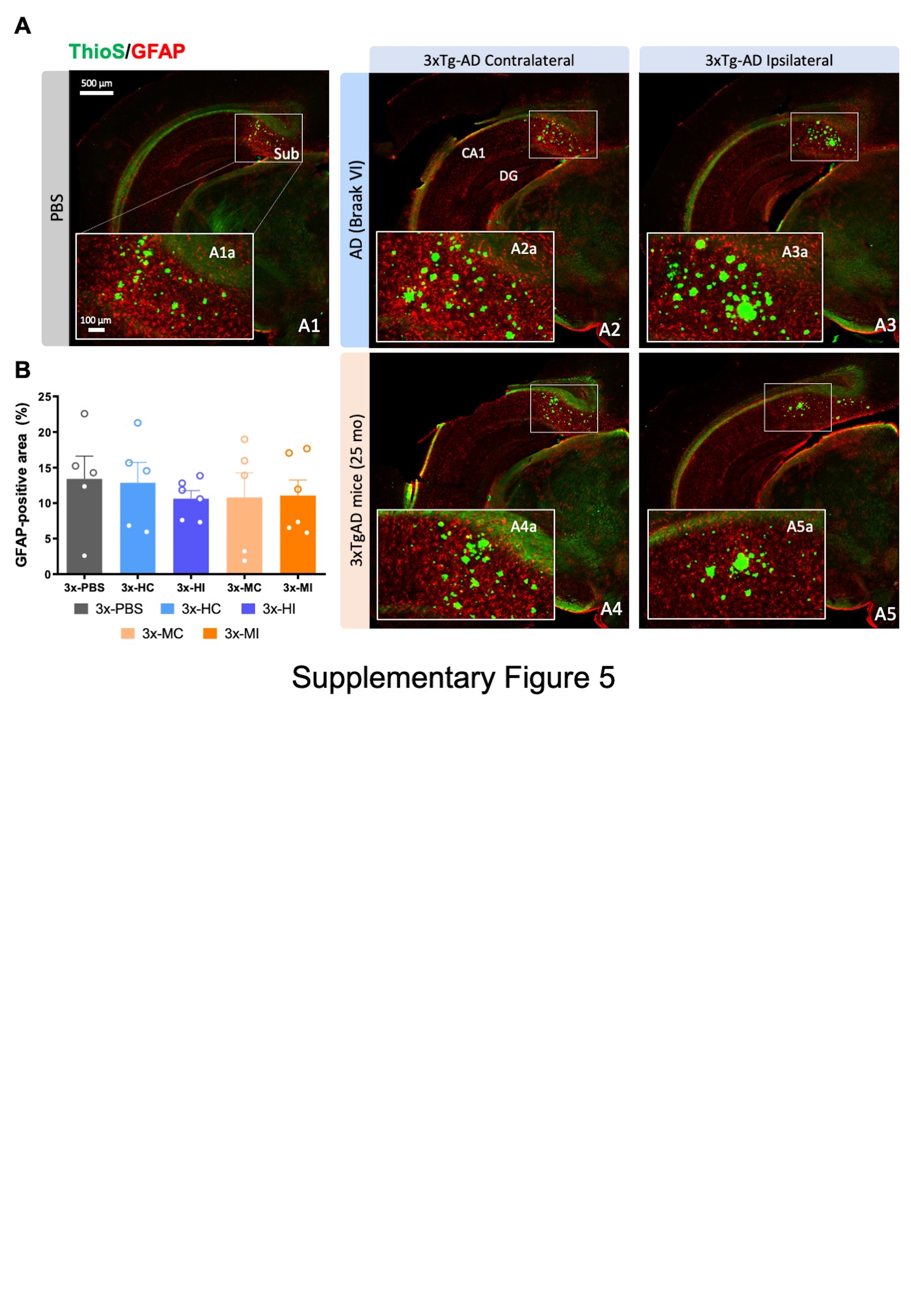

Supplement: Supplementary file 5 — Figure S5. [file ACEL-24-e70094-s005.jpeg]

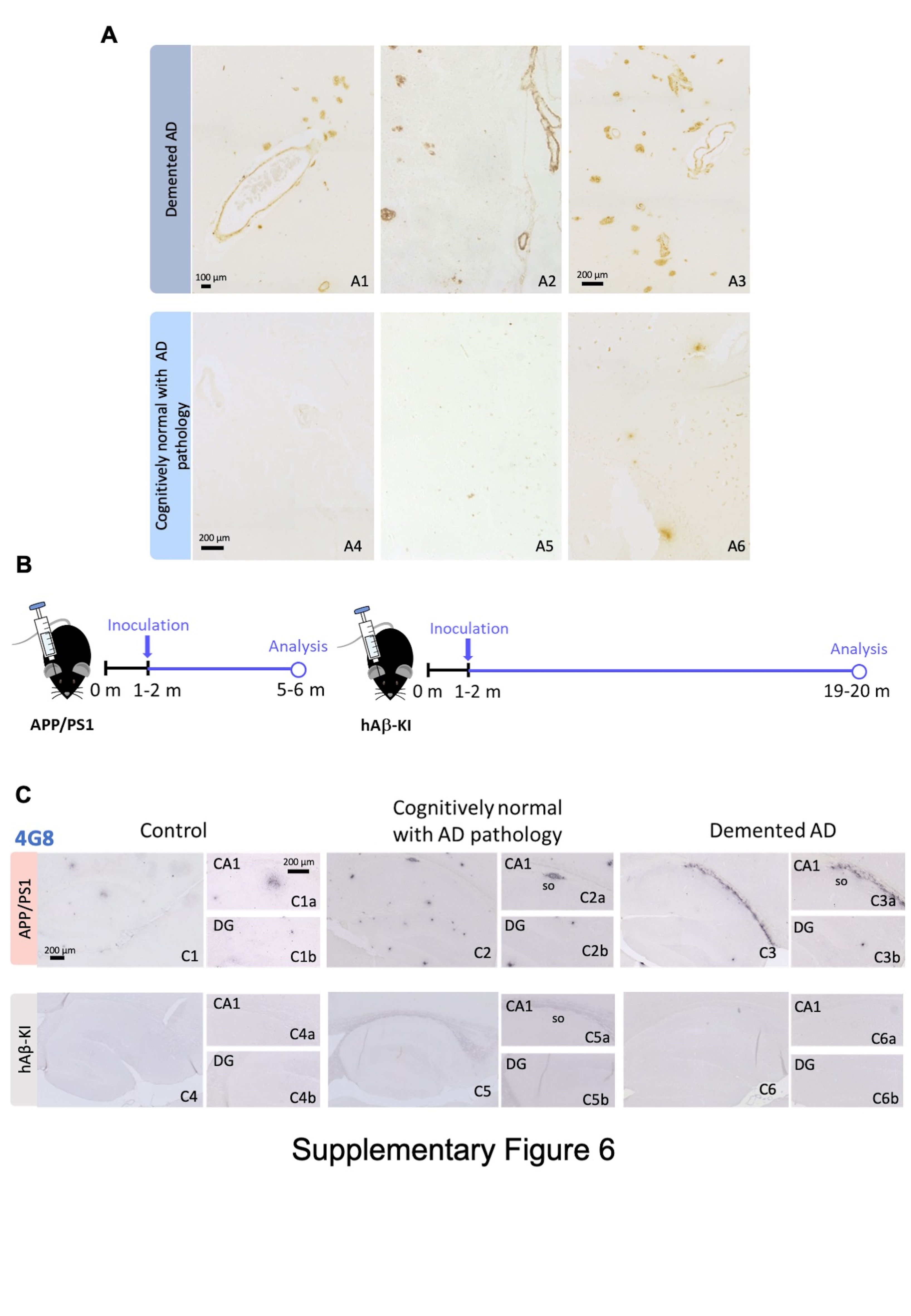

Supplement: Supplementary file 6 — Figure S6. [file ACEL-24-e70094-s002.jpeg]
